# Positron emission tomography imaging biomarker and artificial intelligence for the characterization of solitary pulmonary nodule

**DOI:** 10.3389/fnume.2025.1611823

**Published:** 2025-07-04

**Authors:** Ashish Kumar Jha, Umeshkumar Baburao Sherkhane, Nilendu C. Purandare, Leonard Wee, Andre Dekker, Venkatesh Rangarajan

**Affiliations:** ^1^ Department of Nuclear Medicine and Molecular Imaging, Tata Memorial Hospital, Mumbai, Maharashtra, India; ^2^ Homi Bhabha National Institute, BARC Training School Complex, Mumbai, Maharashtra, India; ^3^ Department of Radiation Oncology (Maastro), University Maastricht School of Oncology and Maastricht University Medical Centre+, Maastricht, Netherlands

**Keywords:** lung cancer, SPN, pet scan, radiomics, random forest algorithm, SMOTE, cross-validation, bootstrap

## Abstract

**Background:**

The characterization of solitary pulmonary nodules (SPNs) as malignant or benign remains a diagnostic challenge using conventional imaging parameters. The literature suggests using combined Positron Emission Tomography (PET) and Computed Tomography (CT) to characterise a SPN. Radiomics and machine learning are other promising technologies which can be utilised to characterise the SPN.

**Purpose:**

This study explores the potential of PET radiomics signatures and machine learning algorithms to characterise the SPN.

**Methods:**

This retrospective study aimed to characterize solitary pulmonary nodules (SPNs) using PET radiomics. A total of 163 patients who underwent PET/CT imaging were included in this study. A total of 1,098 features were extracted from PET images using PyRadiomics. To optimize model performance two strategies i.e., (a) feature selection and (b) feature reduction techniques were employed, including hierarchical clustering, RFE in feature selection, and PCA in feature reduction. To address outcome class imbalance, the dataset was statistically resampled (SMOTE). A random forest models was developed using original training set (RF-Model-O & RF-PCA-Model-O) and balanced training dataset (RF-Model-B & RF-PCA-Model-B) and validated on the test datasets. Additionally, 5-fold cross-validation and bootstrap validation was also performed. The model's performance was assessed using various metrics, such as accuracy, AUC, precision, recall, and F1-score.

**Results:**

Of the 163 patients (aged 36–76 years, mean age 58 ± 7), 117 had malignant disease and 46 had granulomatous or benign conditions. In **Strategy (a),** five radiomic features were identified as optimal using hierarchical clustering and RFE. In **Strategy (b),** five principal components were deemed optimal using PCA. The model accuracy of RF-Model-O and RF-Model-B in the train-test validation, 5-fold cross-validation and bootstrap validation were found to be 0.8, 0.80 ± 0.07, 0.84 ± 1.11 and 0.8, 0.83 ± 0.10, 0.80 ± 0.07 in Strategy (a). Similarly, the model accuracy of RF-PCA-Model-O and RF-PCA-Model-B in the train-test validation, 5-fold cross-validation and bootstrap validation were found to be 0.84, 0.80 ± 0.07, 0.84 ± 07 and 0.74, 0.80 ± 0.08, 0.75 ± 0.08 in Strategy (b).

**Conclusion:**

The PET radiomics demonstrated excellent performance in characterizing SPNs as benign or malignant.

## 1 Introduction

The detection and malignancy characterization of solitary pulmonary nodules (SPNs) represent a significant challenge in the realm of modern healthcare, particularly within the field of radiology (1). An SPN is defined as a rounded or oval lesion that measures up to 3 cm in diameter, entirely surrounded by lung parenchyma, and lacks any associated lymphadenopathy or atelectasis (1). SPNs can be either benign or malignant, and distinguishing between these two categories is critical for appropriate patient management (2, 3). With lung cancer being one of the leading causes of cancer-related deaths worldwide, early and accurate identification of SPNs becomes paramount in improving patient outcomes and reducing mortality rates (4, 5).

In earlier days chest radiographs were used to detect SPN (6–8). In recent years, the landscape of SPN detection has undergone a transformative shift, largely attributed to the advancements in radiological imaging techniques. Imaging modalities such as chest radiography, computed tomography (CT), positron emission tomography (PET), and magnetic resonance imaging (MRI) have demonstrated remarkable capabilities in enhancing the accuracy and efficiency of SPN detection (9–17). The integration of these imaging technologies has revolutionized the approach to identifying SPNs, enabling clinicians to make well-informed decisions about patient management and treatment strategies (9–17).

Traditional chest radiography serves as the initial screening tool for detecting SPNs (6, 7). However, its limited sensitivity often results in a substantial number of SPNs remaining undetected (6, 7). While chest radiography may identify larger nodules, its efficacy diminishes with smaller lesions, emphasizing the need for more advanced imaging techniques (6, 7).

Computed Tomography (CT) has emerged as a cornerstone in SPN detection and characterization due to its exceptional spatial resolution. High-resolution CT (HRCT) plays a pivotal role in distinguishing between benign and malignant SPNs, allowing radiologists to analyze nodule morphology, texture, and enhancement patterns (9–13). The introduction of multidetector-row CT (MDCT) further enhances image quality and facilitates three-dimensional reconstructions, enabling precise measurements of nodule size and volume. CT is particularly advantageous for assessing nodules in difficult-to-reach anatomical locations or those obscured by surrounding structures (9–14).

Positron Emission Tomography (PET) imaging, often combined with CT (PET/CT), offers metabolic and anatomical information simultaneously (15–19). By using radiopharmaceuticals labelled with positron-emitting isotopes, such as Fluorine-18 fluorodeoxyglucose (F-18 FDG), PET detects areas of increased glucose metabolism, which is characteristic of many malignant tumours. PET/CT can aid in differentiating benign from malignant SPNs by assessing their metabolic activity (15–19). For instance, increased FDG uptake suggests malignancy, whereas reduced uptake is indicative of benignity. This modality contributes significantly to staging, guiding biopsy, and monitoring treatment response.

Magnetic Resonance Imaging (MRI) although less frequently employed for SPN detection due to its relatively lower resolution and longer acquisition times, MRI offers unique advantages, especially in assessing nodules in specific clinical scenarios (20–22). MRI provides excellent soft tissue contrast, which can aid in differentiating between various tissue types. Diffusion-weighted imaging (DWI) is particularly promising, as it captures variations in the diffusion of water molecules, highlighting differences in tissue cellularity and microstructure (20–22).

The integration of these radiological imaging modalities into clinical practice has considerably enhanced the accuracy of SPN detection and characterization. However, challenges persist in distinguishing between benign and malignant SPNs, particularly in cases where imaging features are inconclusive (23–25). To address this, various quantitative imaging techniques and computer-aided diagnostic (CAD) systems have been developed, aiming to provide more objective and reproducible assessments of SPNs. These approaches involve the extraction of quantitative features from imaging data, such as nodule size, shape, texture, and enhancement patterns, which are then used to develop predictive models to aid radiologists in their decision-making process (26–31).

The detection and characterization of solitary pulmonary nodules have seen remarkable advancements through the integration of various radiological imaging modalities and artificial intelligence (27–31). From the initial screening with chest radiography to the high-resolution capabilities of CT, the metabolic insights of PET/CT, and the unique tissue contrast of MRI, radiology-based radiomics and artificial intelligence may have an indispensable role in improving the accuracy and efficacy of SPN diagnosis (27–32). These technologies have transformed the clinical landscape, facilitating early detection, accurate characterization, and appropriate management of SPNs. Continued research and innovation in this field hold the promise of further refining our understanding and approach to SPN detection, ultimately leading to improved patient outcomes and reduced mortality rates (24, 25). In this study, we utilized AI and PET radiomics to differentiate between malignant and benign etiologies of solitary pulmonary nodules (SPNs).

## 2 Material and methods

This retrospective study was approved by the Institutional Ethics Committee (IEC), with a waiver of informed consent granted in accordance with the Declaration of Helsinki. This study adheres TRIPOD Checklist for prediction model development (Supplementary TRIPOD Checklist). The study included 196 patients who underwent PET/CT imaging for characterization of solitary pulmonary nodules (SPNs) between 2016 and 2020. Patients in this study were selected based on the following inclusion and exclusion criteria:

**Inclusion criteria:**

Patients enrolled between 2000 and 2016.
Patients who underwent PET/CT scans.
Patients with a histopathology-proven diagnosis.

**Exclusion criteria:**

PET/CT scans of suboptimal quality.
Non-conclusive histopathology reports.

**Figure 1:**
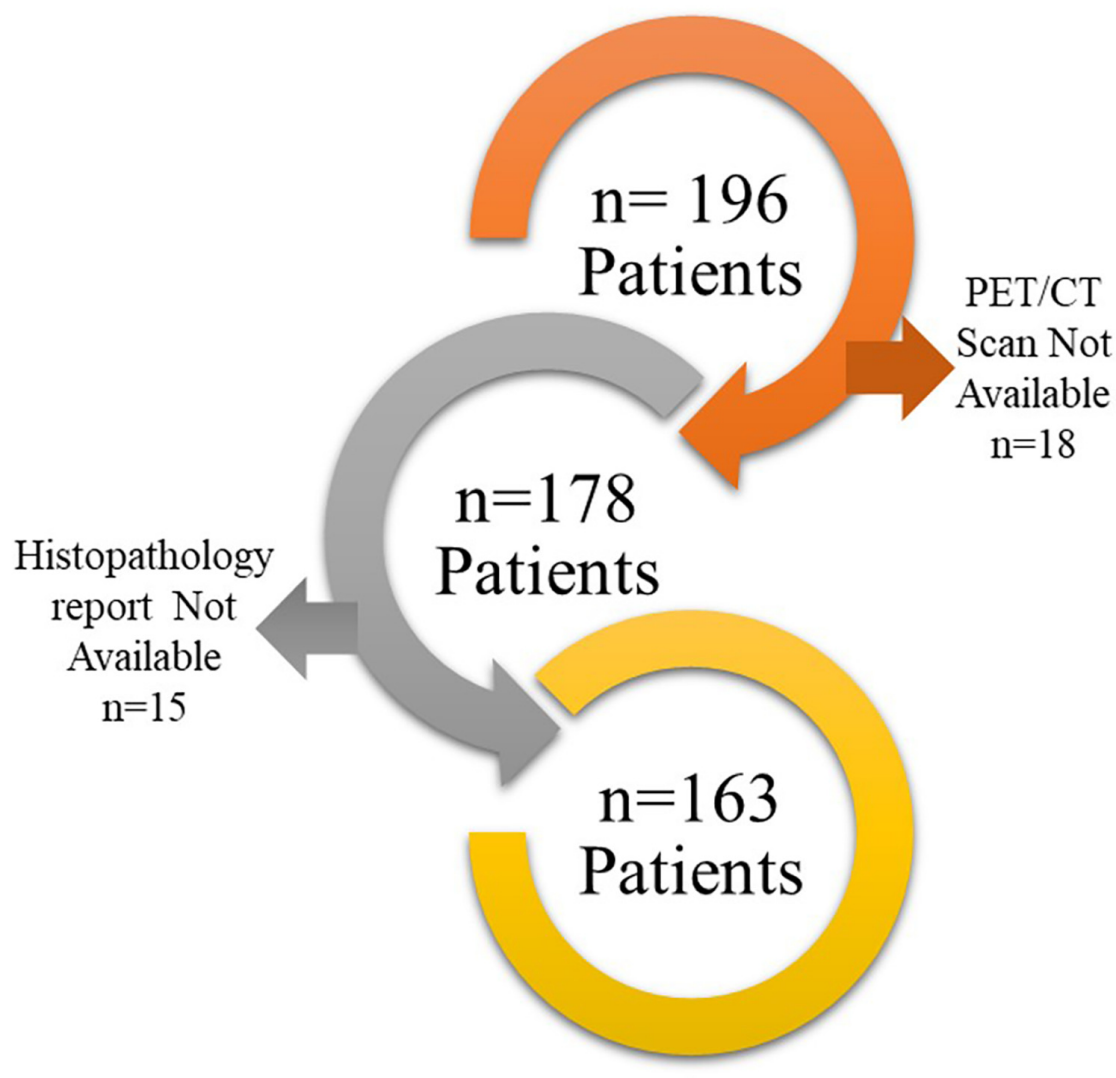
Patient selection flowchart is shown in the figure.

Finally, 163 patients were found suitable to include in this study (Figure 1). PET/CT scans of these patients were downloaded from the Picture Archive and Communication System (PACS) to EBW workstation, Phillips Medical system, Eindhoven, Netherlands and the nodules were delineated by an expert radiologist using the SUV threshold method (threshold = 42%) followed by manual corrections if required. Manual corrections were required for tumor with an SUVmax less than 3 g/ml. such correction was performed by an expert radiologist with more than 20 years of experience. The delineation was stored as RTSTRUCT by the name of GTV in Digital Image Communication in Medicine (DICOM) format. The maximum Standardized Uptake Value (SUVmax) in the delineation was also captured and stored in the datasheet. Subsequently, PET scan images and GTV were transferred to the AI workstation for further processing.

All the patients having malignant SPN were treated with surgery as first-line treatment. Patients diagnosed with granulomatous disease were referred to the general hospital for further treatment.

### 2.1 PET/CT acquisition

The contrast-enhanced CT scan followed by PET scans were performed one hour after the intravenous administration of 18F-FDG (5 MBq/kg body weight) on Gemini TF16 or Gemini TF 64 PET/CT scanner, Phillips Medical system, Eindhoven, Netherlands. The details of imaging parameters are shown in Table 1.

**Table 1:** Imaging protocols used to perform PET/CT scan.

PET	Acquisition mode	List mode
	Matrix size	200 × 200
	Bed position (sec)	90
	Attenuation correction	CT based
	Reconstruction	RAMLA
CT	kVp	120
	mAs	200
	Slice thickness (mm)	5
	Tube rotation time (sec)	0.5
	Matrix size	512 × 512
	Reconstruction	ASiR

### 2.2 Prediction model development

**Figure 2:**
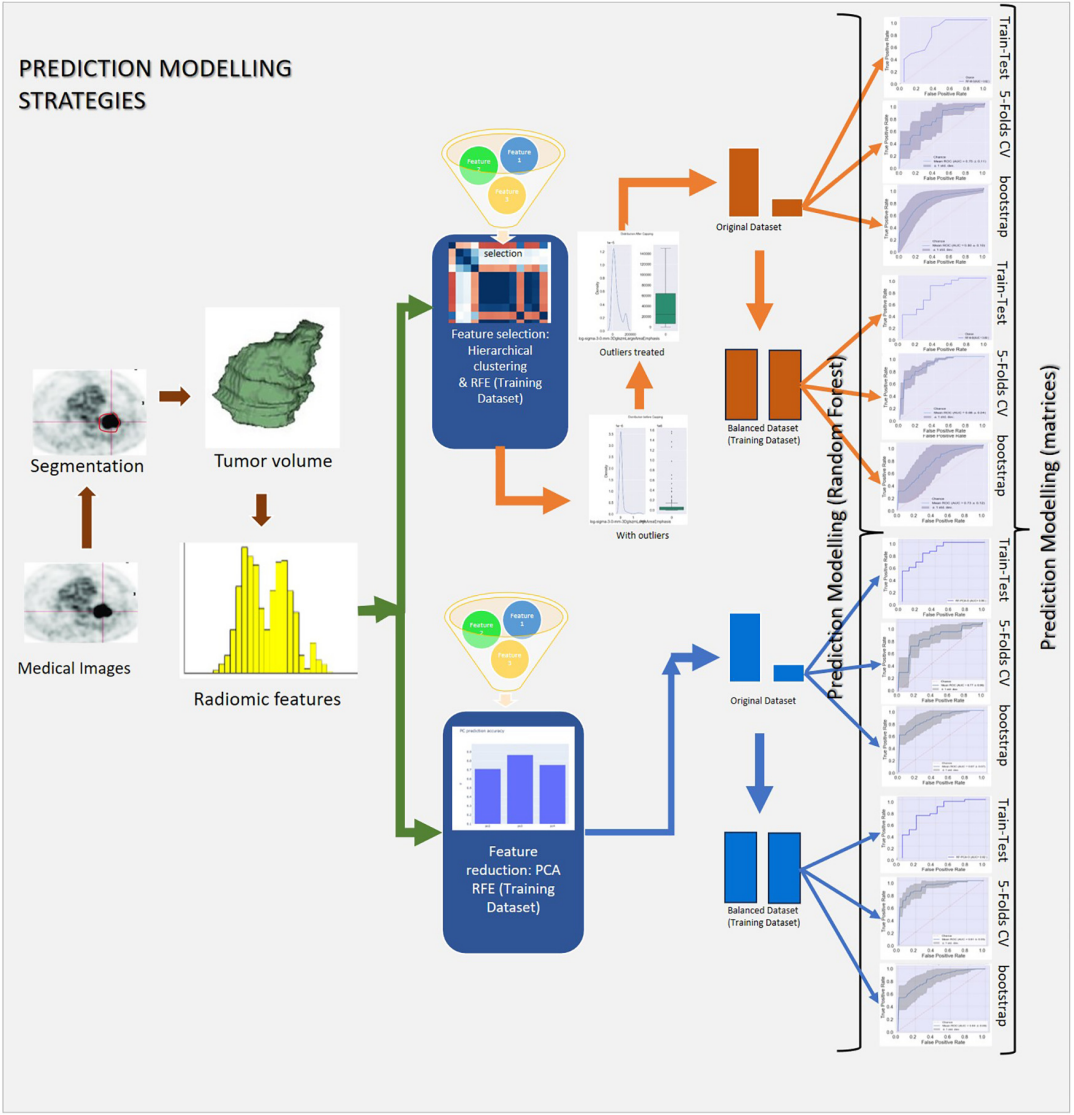
Schematic representation of the study's methodology, outlining data preprocessing, hyperparameter tuning, model development, and validation.

The complete prediction modelling strategy is pictorially depicted in Figure 2. As the first step of the radiomics-based prediction model development, 1,098 features were extracted from the PET images and GTV by using the in-house developed radiomic workflow software PyRadGUI (33). A description of the extracted radiomic feature types is presented in this table (Table 2). To improve the model's predictive performance, we implemented two feature engineering strategies: (a) feature selection and (b) feature reduction.

**Table 2:** The table outlines the various types of radiomic features extracted from the images.

Type of Feature	Feature descriptions	No. of Features
Shape based Features	Independent of the gray level intensity distribution, shape features characterize the three-dimensional size and shape of the ROI. Their calculation relies solely on the original image and mask.	13
First Order statistics	First-order statistics describe the distribution of voxel intensities within the ROI.	17
GLRLM	The Gray Level Run Length Matrix (GLRLM) assesses the distribution of run lengths for discretized grey levels in an image or a stack of images.	16
GLCM	The Gray Level Co-Occurrence Matrix (GLCM) characterizes how often specific combinations of discretized intensities occur for neighboring voxels within a 3D volume, distributing this information along various image directions.	22
GLSZM	The Gray Level Size Zone Matrix (GLSZM) measures the distribution of different-sized zones, where each zone consists of connected voxels with the same discretized grey level.	16
NGTDM	The Neighboring Gray Tone Difference Matrix (NGTDM) represents a sum derived from comparing each voxel's discretized gray level to the average discretized gray level of its neighbors, considering voxels within a Chebyshev distance *δ*.	5
GLDM	The Gray Level Dependence Matrix (GLDM) assesses the relationships between a central voxel and its neighboring voxels by counting how many connected neighbors, within a distance δ, share a similar or “dependent” gray level.	14
LoG Features	Application of a Laplacian of Gaussian (LoG) filter to the original image yields a set of derived images for each designated sigma value. While 1–5 sigma values are commonly employed, our analysis utilized three sigma values (1, 2, and 3), resulting in three corresponding sets of derived images. Radiomic features are then extracted from these resultant image sets.	270
Wavelet Features	Wavelet transformation is applied to the original image set using three-dimensional wavelet decomposition, generating eight derived image sets. Radiomic features are then extracted from these transformed sets.	720

#### 2.2.1 Feature selection

A two-step feature selection process was adopted to select the most relevant features for the prediction model development.

##### 2.2.1.1 Unsupervised learning technique

K-means Clustering was performed to reduce the redundancy in features based on their similarity. By identifying clusters of correlated features, we could potentially mitigate redundancy and select representative features from each cluster.

##### 2.2.1.2 Recursive feature elimination (RFE)

RFE is a backward feature elimination method that iteratively removes features that have the least impact on the model's performance. This helps to identify the most informative features for the prediction task. Using the Random forest algorithm in the RFE algorithm the most significant features were selected to develop a prediction model.

#### 2.2.2 Feature reduction

##### 2.2.2.1 Principal component analysis (PCA)

The PCA is a dimensionality reduction technique which, transforms the original features into a new set of uncorrelated features, known as principal components. By retaining only the most important principal components, we can significantly reduce the dimensionality of the data while preserving the most relevant information. The PCA was performed to select the most significant principal components for the prediction model development.

The outliers in the radiomics features may influence the performance of radiomic-based prediction model.

#### 2.2.3 Outlier treatment

The interquartile range method was employed to identify and treat the outliers in the selected features. The values falling outside the 1.5 times the interquartile range were considered outliers. The outliers were replaced with the median or mean value randomly to prevent them from unduly influencing the model's performance. Outliers treatment was performed accordingly for 10 radiomic feature selected by using k-mean clustering and SUV max value (Supplementary Figures S1–S10).

#### 2.2.4 Train-test data split

The stratified train-test dataset split was performed in a 7:3 ratio. 70% of data were used to train the model and 30% were used to validate the model subsequently. The training dataset underwent feature selection via recursive feature elimination (RFE), data balancing, and hyperparameter tuning. In contrast, the test dataset was reserved solely for model validation in train-test validation and bootstrap validation, ensuring an unbiased evaluation of the model's performance and data leakage.

#### 2.2.5 Data balancing

Given the class imbalance in the dataset, with a higher prevalence of malignancies compared to benign SPNs, we implemented data balancing techniques. The SMOTE (Synthetic Minority Oversampling Technique) technique was used to address the class imbalance by generating synthetic data points for the minority class in the training dataset (33). By creating new samples similar to existing minority classes, SMOTE helps to balance the representation of different classes in the training dataset.

#### 2.2.6 Prediction algorithm

Random Forest Algorithm: The random forest (RF) algorithm was employed to develop the prediction model. RF is a popular ensemble machine learning algorithm that combines multiple decision trees to make predictions. Random forests are known for their robustness and ability to handle complex relationships in data.

#### 2.2.7 Hyperparameter tuning

A grid search method was employed for exhaustively searching through a predefined grid of hyperparameter values to find the optimal combination of these hyperparameters by fine-tuning them, which maximizes the prediction model performance of the prediction model by in the model. By systematically evaluating different parameter settings, grid search helps to fine-tune the model's behaviour and improve its predictive accuracy (Supplementary Table S3).

In strategy one two random forest prediction models i.e., RF-Model-O and RF-Model-B were developed using original and balanced train datasets. In strategy two, two models i.e., RF-PCA-Model-O and RF-PCA-Model-B were developed using original and balanced train datasets. These models were validated using the test dataset. To assess the prediction model's performance and generalization capabilities, we employed five-fold cross-validation and bootstrap validation in addition to the standard train-test split.

### 2.3 Prediction metrics

The performance of the prediction models was evaluated using various metrics, including accuracy, AU-ROC (Area Under the Receiver Operating Characteristic Curve), precision, recall, and F1-score. These metrics provide insights into the model's ability to correctly classify instances, avoid false positives and negatives, and achieve a balance between precision and recall.

### 2.4 Software used

All statistical analyses and prediction model development were performed using various packages of Python version 3.10. For feature selection and prediction model development scikit-learn 1.6.0 package of python was used.

## 3 Results

A total of 163 patients had a mean age of 59.5 ± 12.5 (Range: 36–76) years were included in this study. Of 163 patients, 117 had malignancy and 46 had granulomatous or benign disease. The mean lesion size was 3 ± 1.6 cubic centimetres. The mean SUVmax was found to be 10.2 ± 6.1 g/ml. the details of the demography data are provided in Table 3.

**Table 3:** Demographic and clinical characteristics of study participants.

Sample size (n)	163
Biological Sex	
Male	116
Female	47
Smoker	99
Non-smoker	64
Age (years)	59.5 ± 12.5 (Range: 36–76)
Lesion volume (cc)	3 ± 1.6 cc
Lesion size (largest diameter)	2.15 cm (range: 0.6–3 cm)
	Size of 80% lesions was >2 cm
Mean SUVmax (g/ml)	10.2 ± 6.1 g/ml (Range: 1.1–18.7 g/ml)
Pathology	*n*
Malignant	117
Adenocarcinoma	71
Squamous cell carcinoma	25
Adenocarcinoma *in situ* (BAC)	05
Low-grade neuroendocrine carcinoma	16
Benign	46
Tuberculosis	16
Nonspecific inflammation	24
Fungal	02
Sclerosing hemangioma	02
Chondroid hamartomas	02
Total	163

**Figure 3:**
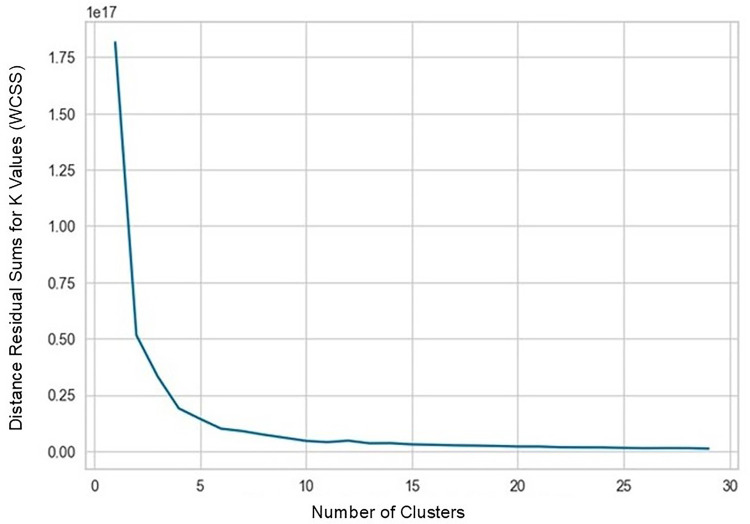
K-Means clustering elbow curve, indicating 10 optimal clusters.

**Figure 4:**
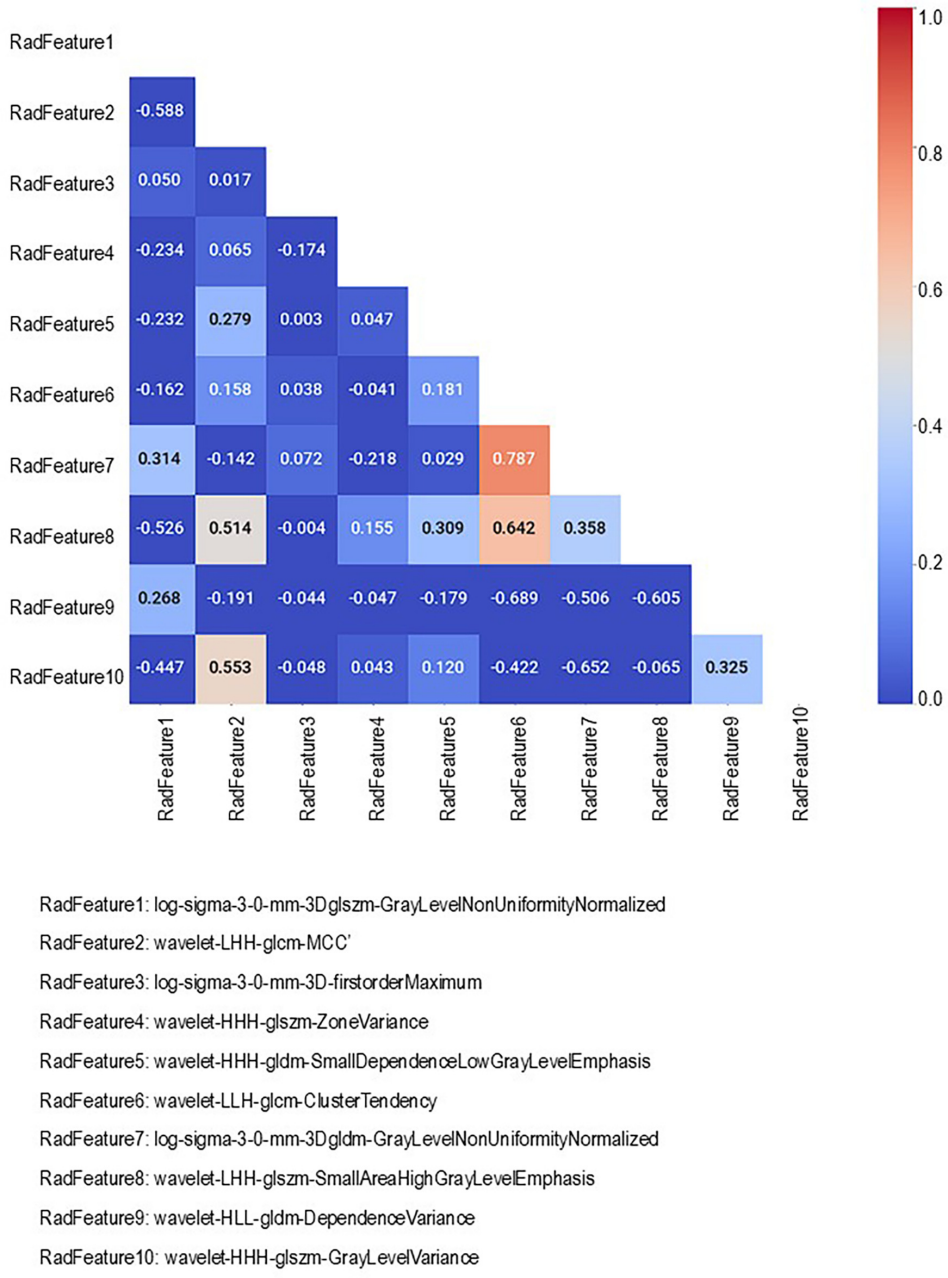
Spearman correlation heatmap of 10 radiomic features identified via K-means clustering.

**Figure 5:**
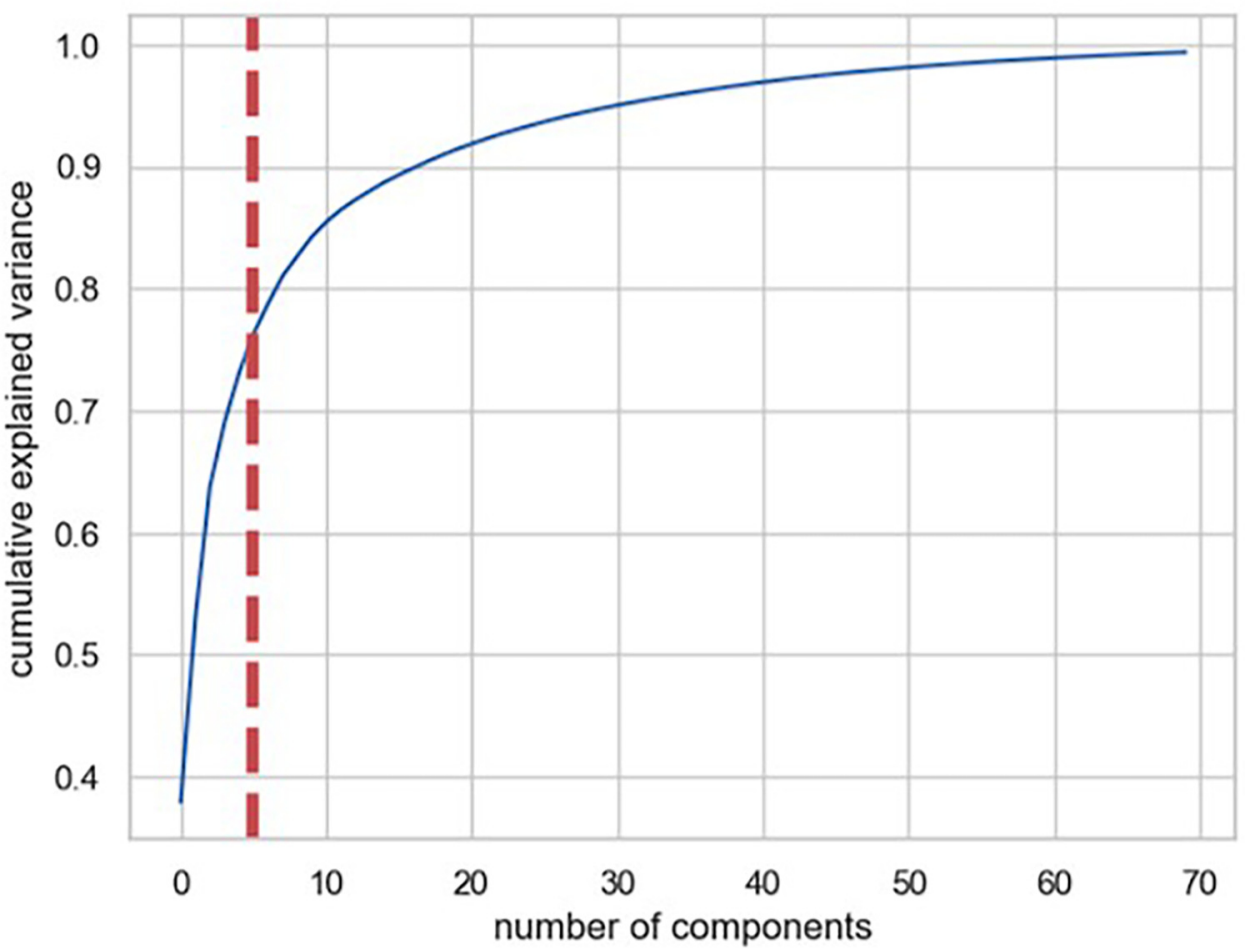
Figure shows the commutative variance ration of principal components.

In strategy (a), Spearman correlation-based K-mean Clustering yielded 10 features (Figure 3) (Supplementary Tables S1, S2). The heatmap of the Spearman correlation plot of 10 features is shown in Figure 4. Finally, 5 optimal features were selected using recursive feature elimination which was used for the prediction model development using a random forest algorithm (Supplementary Table S3). In strategy (b), 5 principal components were found to be optimal for prediction model development using random forest algorithm (Figure 5 & Supplementary Table S4).

**Figure 6:**
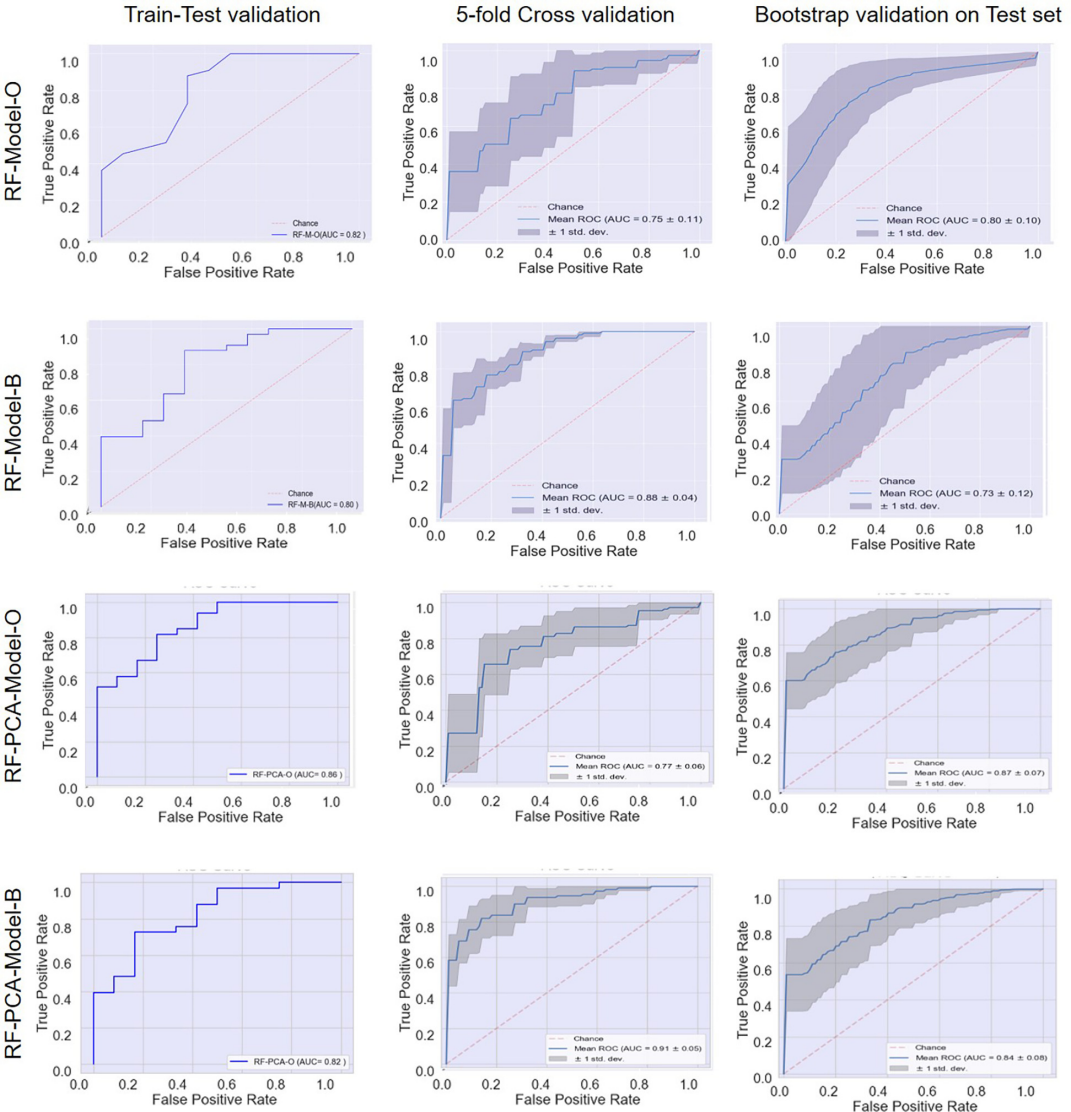
Receiver operating characteristic (ROC) curves: model performance comparison using train-test, 5-fold cross-validation, and bootstrap validation.

In strategy a), the accuracy of the RF-Model-O (random forest model) was found to be 0.8, 0.80 ± 0.07, and 0.84 ± 1.11 on the test set, 5-fold cross-validation and bootstrap validation respectively on the original dataset. Similarly, the RF-Model-B model accuracy was found to be 0.8, 0.83 ± 1.10, and 0.80 ± 0.07 on the test set, 5-fold cross-validation and bootstrap validation respectively on balancing the test dataset. The RF-PCA-Model-O model accuracy using the PCA feature selection method was found to be 0.84, 0.80 ± 0.07 and 0.84 ± 0.07 on the test set, cross-validation and bootstrap validation on test set. Similarly, the RF-PCA-Model-B model accuracy using the PCA feature selection method was found to be 0.74, 0.80 ± 0.00 and 0.75 ± 0.08 on the test set, cross-validation and bootstrap validation on test set. The detailed prediction matrices of the random forest model are shown in Table 4 (Supplementary Figures S11, 12 and Supplementary Tables S5, S6). The AUC of ROC curves of all the prediction models in validation are shown in Figure 6.

**Table 4:** Prediction matrices show the performance of various models in train-test, 5-fold cross-validation and bootstrap validation.

Prediction algorithm	Feature Selection	Data/Model	Validation	Accuracy	Precision	Recall	f1-score	AUC of ROC
Random Forest Model	Hierarchical clustering and REF	Original/RF-Model-O	Train-Test	0.80	0.81	0.80	0.80	0.79
			5-folds CV	0.80 ± 0.07	0.85 ± 0.07	0.86 ± 0.06	0.87 ± 0.07	0.75 ± 0.11
			Bootstrap	0.84 ± 1.11	0.84 ± 0.07	0.91 ± 0.05	0.87 ± 0.04	0.80 ± 0.10
		Balanced/RF-Model-B	Train-Test	0.80	0.80	0.80	0.80	0.79
			5-folds CV	0.83 ± 1.10	0.79 ± 0.04	0.80 ± 0.07	0.79 ± 0.06	0.88 ± 0.07
			Bootstrap	0.80 ± 0.07	0.85 ± 0.07	0.88 ± 0.05	0.86 ± 0.05	0.73 ± 0.12
	PCA	Original/RF-PCA-Model-O	Train-Test	0.84	0.86	0.93	0.89	0.86
			5-folds CV	0.80 ± 0.07	0.81 ± 0.08	0.86 ± 0.08	0.83 ± 0.04	0.77 ± 0.06
			Bootstrap	0.84 ± 0.07	0.86 ± 0.08	0.94 ± 0.07	0.89 ± 0.05	0.87 ± 0.07
		Balanced/RF-PCA-Model-B	Train-Test	0.74	0.90	0.70	0.79	0.82
			5-folds CV	0.80 ± 0.08	0.80 ± 0.10	0.75 ± 0.09	0.77 ± 0.10	0.91 ± 0.05
			Bootstrap	0.75 ± 0.08	0.94 ± 0.06	0.71 ± 0.09	0.80 ± 0.06	0.84 ± 0.08

## 4 Discussion

Lung cancer remains one of the most prevalent and deadliest malignancies worldwide, posing significant challenges for healthcare providers. The timely and accurate detection of lung cancer is paramount to improving patient outcomes, particularly in the case of solitary pulmonary nodules (SPNs) (34). A solitary pulmonary nodule is defined as a discrete, well-circumscribed lesion in the lung parenchyma, often detected incidentally in imaging studies, which raises concerns about potential malignancy (34). While SPNs can be benign, they also represent an important clinical dilemma due to the potential for malignancy (34). Therefore, distinguishing between benign and malignant SPNs is a critical challenge that necessitates precise and efficient diagnostic tools.

This study retrospectively analysed 163 patients with solitary pulmonary nodules (SPN) upon presentation, 117 of whom had histologically proven malignant disease. We employed PET radiomics-based prediction modelling using the well-established Random Forest algorithm. Our model demonstrated excellent discriminatory capabilities in identifying malignant and benign lesions, achieving a validation accuracy of 0.8 and an AUC of 0.9.

The advent of advanced imaging modalities, such as computed tomography (CT), has revolutionized the early detection and diagnosis of lung nodules. However, the sheer volume of medical images generated has made manual interpretation impractical, leading to the emergence of computer-aided detection (CAD) and diagnosis (CADx) systems (35–37). These systems integrate machine learning, deep learning, and artificial intelligence (AI) algorithms to enhance the diagnostic accuracy and efficiency of radiologists. By analyzing vast amounts of image data and identifying subtle patterns indicative of malignancy, these systems hold the potential to significantly improve SPN detection rates (37).

Despite the promising potential of CAD and CADx systems, several challenges persist in the accurate detection of SPNs. One key challenge is the variability in SPN appearances, encompassing diverse morphologies, sizes, and locations within the lung. The complexity of distinguishing malignant nodules from benign ones requires a high level of precision in algorithm design and training (37–39). Moreover, in the last few years, it has been reported that the role of functional imaging like PET/CT is increasing in SPN detection. The function imaging-based glucose metabolic markers like SUV characterize the SPN better than morphological parameters on CT or MRI scans (31). A study performed by Purandere et al. demonstrated the improved accuracy of SPN detection based on SUVmax obtained from metabolic imaging of 18F-FDG (17). Another study by Garcia-Velloso MJ et al. has shown that integrating the metabolic information from 18F-FDG with the morphological information from CT improves the accuracy of lung cancer diagnosis (19). The study by Herder et al. aimed to validate the Swensen clinical prediction model for indeterminate pulmonary nodules and assess the added value of FDG-PET scanning (40). Several studies have suggested that proliferation markers like 18F-FLT can characterize lung cancer better than those of metabolic markers like 18F-FDG (20). A study, by Jha AK et al., was able to predict 2-year overall survival (OS) using the radiomic features extracted from the CT images (41). Several studies have been performed on radiomics to predict various outcomes in lung cancer (33–37, 41, 42). These studies have demonstrated that the heterogeneity in 18F-FDG uptake in tumours carries vital information to predict various outcomes in lung cancer (33–45). In the last few years, many researchers have explored AI-assisted radiomic features to characterize the SPN (37–40, 43–45). Unlike our study, which focuses on radiomics based prediction modelling, Beyer et al. (2007) investigated the practical application of Computer-Aided Detection (CAD) in improving pulmonary nodule detection efficiency (43). Similar to our work, Zhao et al. (2022) described the development of a diagnostic model for malignant solitary pulmonary nodules, though their model utilized CT radiomics features (44). In contrast, our study developed a PET radiomics model, which is more directly based on the physiological manifestation of tumors. In a literature reviews, Thawani et al. provided a broad overview of radiomics and radiogenomics applications in lung cancer (45). However, Wilson et al. focused specifically on the role of radiomics in characterizing pulmonary nodules and lung cancer, discussing how quantitative features from medical images offer insights into tumor biology and aid clinical decision-making (46, 47). These studies have suggested the great role of PET-based radiomics in characterizing the SPN. In this study, we have attempted to characterize SPN using AI-assisted PET radiomic features. We adopted two methods for feature dimensionality reduction i.e., feature selection and feature reduction. In both the methods we found a good accuracy of the model in the characterization of the SPN. We adopted the robust multi-step method of the feature selection that yielded the most significant optimal features for the prediction model development. We employed the regular grid search to tune the hyperparameters of the random forest model. The balancing of the data using SMOTE did not improve the model performance significantly. The accuracy and other prediction matrices of the prediction model developed using feature selection method employing filter method followed by multivariate feature selection using RFE and feature reduction method using PCA method were comparable that shows the robustness of the radiomic feature in characterization of SPNs. The robustness of radiomic-based prediction model is also demonstrated by the 5-fold cross-validation and bootstrap validation as the prediction accuracy and other prediction matrices remain comparable in these methods with that of the train-test method.

These systems can be trained on large datasets of labelled images to learn intricate patterns that are often imperceptible to the human eye. The AI-enhanced detection of SPNs holds great promise not only for improving diagnostic accuracy but also for reducing radiologist fatigue and enhancing workflow efficiency.

To ensure the practicality and clinical applicability of these AI-driven solutions, rigorous validation and testing are imperative. The ability of AI algorithms to generalize across diverse patient populations and imaging devices needs to be thoroughly assessed. Furthermore, these systems should be seamlessly integrated into clinical workflows, taking into consideration regulatory approvals, data privacy, and the need for radiologist oversight. extracting and analyzing quantitative imaging features, radiomics enhances the ability to differentiate between benign and malignant SPNs with greater accuracy than traditional imaging techniques. This non-invasive method allows for more precise risk stratification, guiding clinical decisions regarding biopsy, surveillance, or intervention. Consequently, PET radiomics can improve patient outcomes by reducing unnecessary invasive procedures, optimizing treatment plans, and ultimately facilitating early and accurate diagnosis of lung cancer, thus contributing to better survival rates and quality of life for patients.

This study has several limitations. The first limitation is that the study is a single-centre study. The single-centre study is known to have reduced accuracy in predicting the outcome when tested on external conditions. The second major limitation is the sample size. The robustness of the prediction model increases with an increased sample size. The third major limitation is the imbalanced nature of the dataset. In our study, the malignant SPNs were three times that of benign SPNs. As our hospital is a tertiary cancer care centre so in our dataset, we have a large number of malignant SPN.

Future studies on PET radiomics-based characterization of SPNs could focus on several key areas to further enhance clinical applications and patient outcomes. to conduct multi-center studies to develop and validate the robust radiomic signature. To include CT radiomic features along with PET radiomic features. Prospective validation of radiomic signature in clinical trials. Our future research will involve a multicentric study to validate this prediction model using both retrospective and prospective cohorts.

## 5 Conclusion

The challenges posed by solitary pulmonary nodules are complex and multifaceted and difficult the differentiate between malignant and benign SPNs. The challenge further increased in India due to the prevalence of tuberculosis. This study demonstrates the utility of PET radiomics in the characterization of SPNs. Applying the data balancing technique to the training dataset did not improve the prediction accuracy in this study. By harnessing the power of machine learning algorithms and PET radiomics can bring a transformative shift towards the detection and characterization of SPN. Accurate and efficient SPN detection is vital for timely diagnosis and optimal patient outcomes, particularly in the context of lung cancer.

## Data Availability

The raw data supporting the conclusions of this article will be made available by the authors, without undue reservation.
