# Supplementary material for: Positron emission tomography imaging biomarker and artificial intelligence for the characterization of solitary pulmonary nodule
Source: Front Nucl Med. 2025 Jul 4;5:1611823. doi: 10.3389/fnume.2025.1611823 (PMC12271206; doi:10.3389/fnume.2025.1611823)
Supplement: Supplementary file 1 [file Presentation1.pptx]

## Slide 1
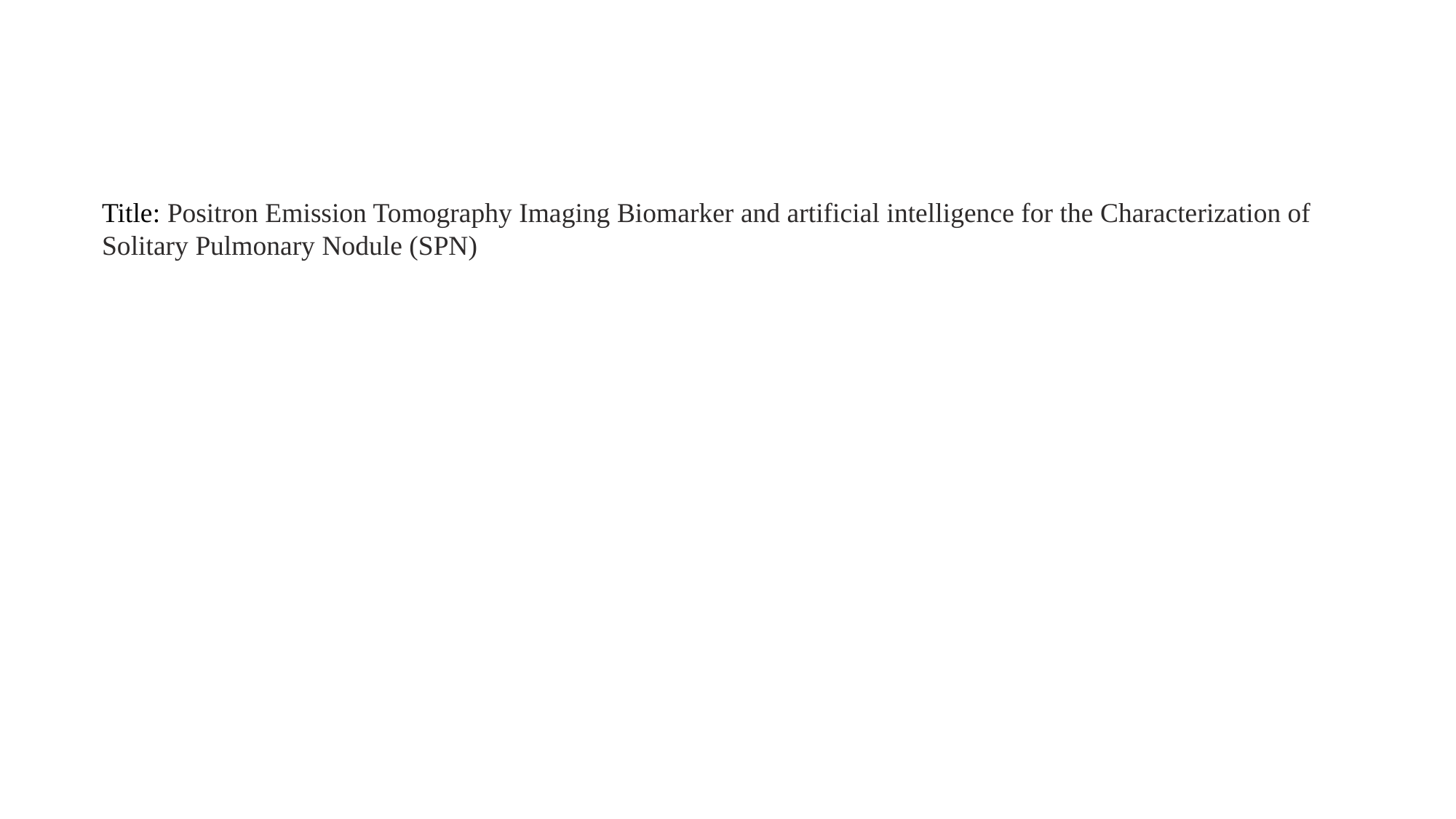

Title: Positron Emission Tomography Imaging Biomarker and artificial intelligence for the Characterization of Solitary Pulmonary Nodule (SPN)

## Slide 2
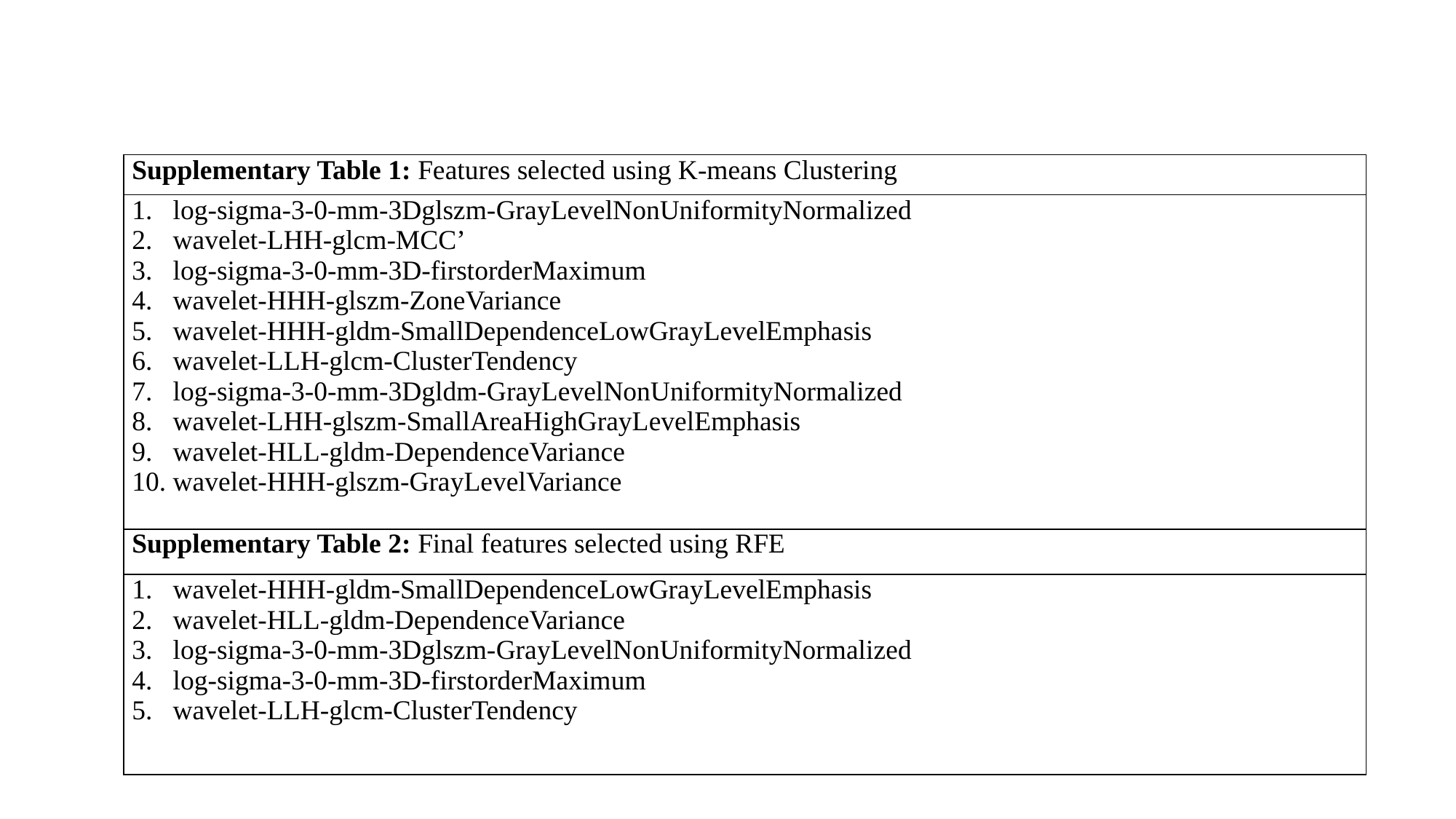

| Supplementary Table 1: Features selected using K-means Clustering |
| --- |
| log-sigma-3-0-mm-3Dglszm-GrayLevelNonUniformityNormalized wavelet-LHH-glcm-MCC’ log-sigma-3-0-mm-3D-firstorderMaximum wavelet-HHH-glszm-ZoneVariance wavelet-HHH-gldm-SmallDependenceLowGrayLevelEmphasis wavelet-LLH-glcm-ClusterTendency log-sigma-3-0-mm-3Dgldm-GrayLevelNonUniformityNormalized wavelet-LHH-glszm-SmallAreaHighGrayLevelEmphasis wavelet-HLL-gldm-DependenceVariance wavelet-HHH-glszm-GrayLevelVariance |
| Supplementary Table 2: Final features selected using RFE |
| wavelet-HHH-gldm-SmallDependenceLowGrayLevelEmphasis wavelet-HLL-gldm-DependenceVariance log-sigma-3-0-mm-3Dglszm-GrayLevelNonUniformityNormalized log-sigma-3-0-mm-3D-firstorderMaximum wavelet-LLH-glcm-ClusterTendency |

## Slide 3
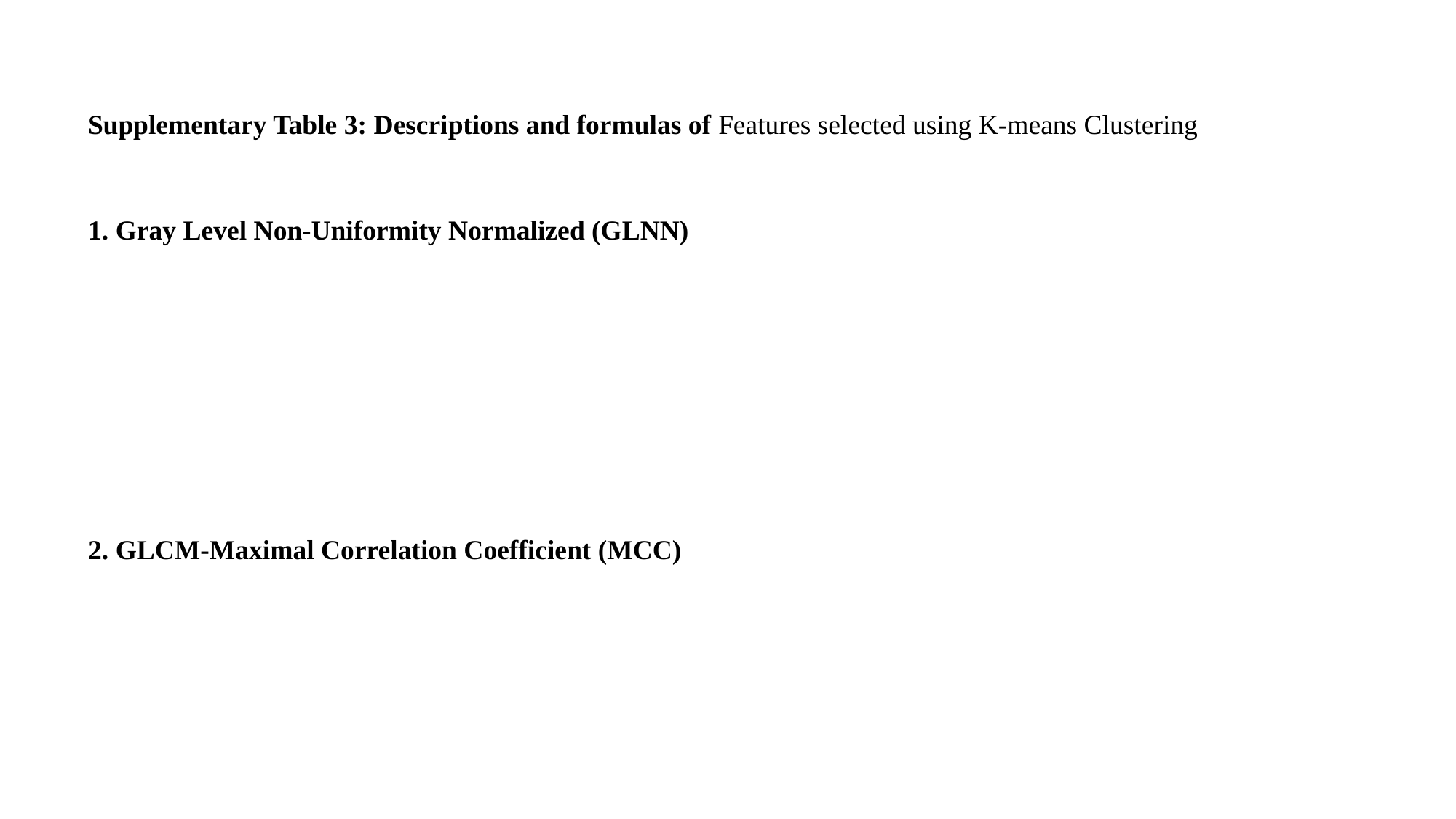

## Slide 4
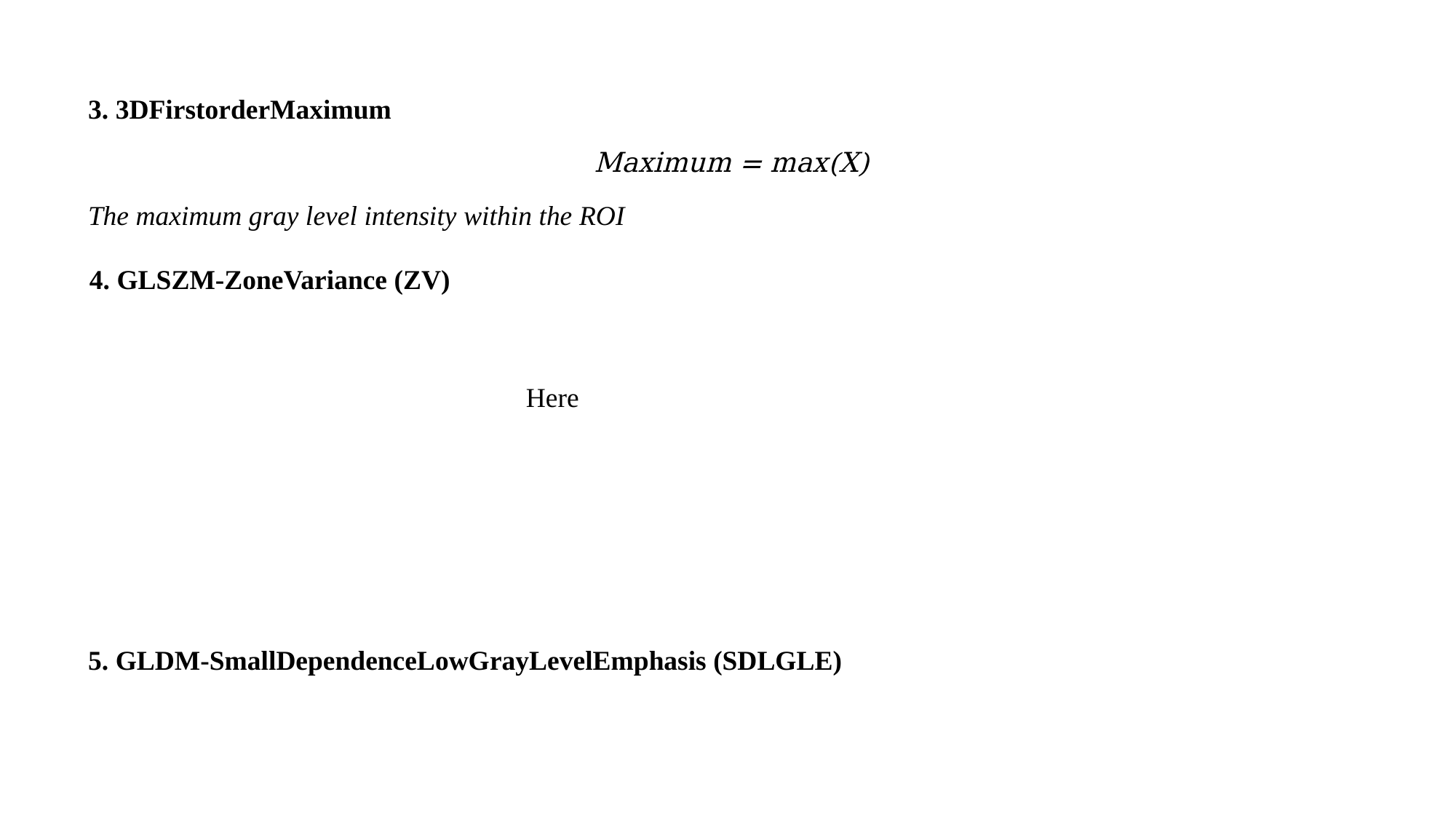

3. 3DFirstorderMaximum
Maximum = max(X)
The maximum gray level intensity within the ROI

## Slide 5
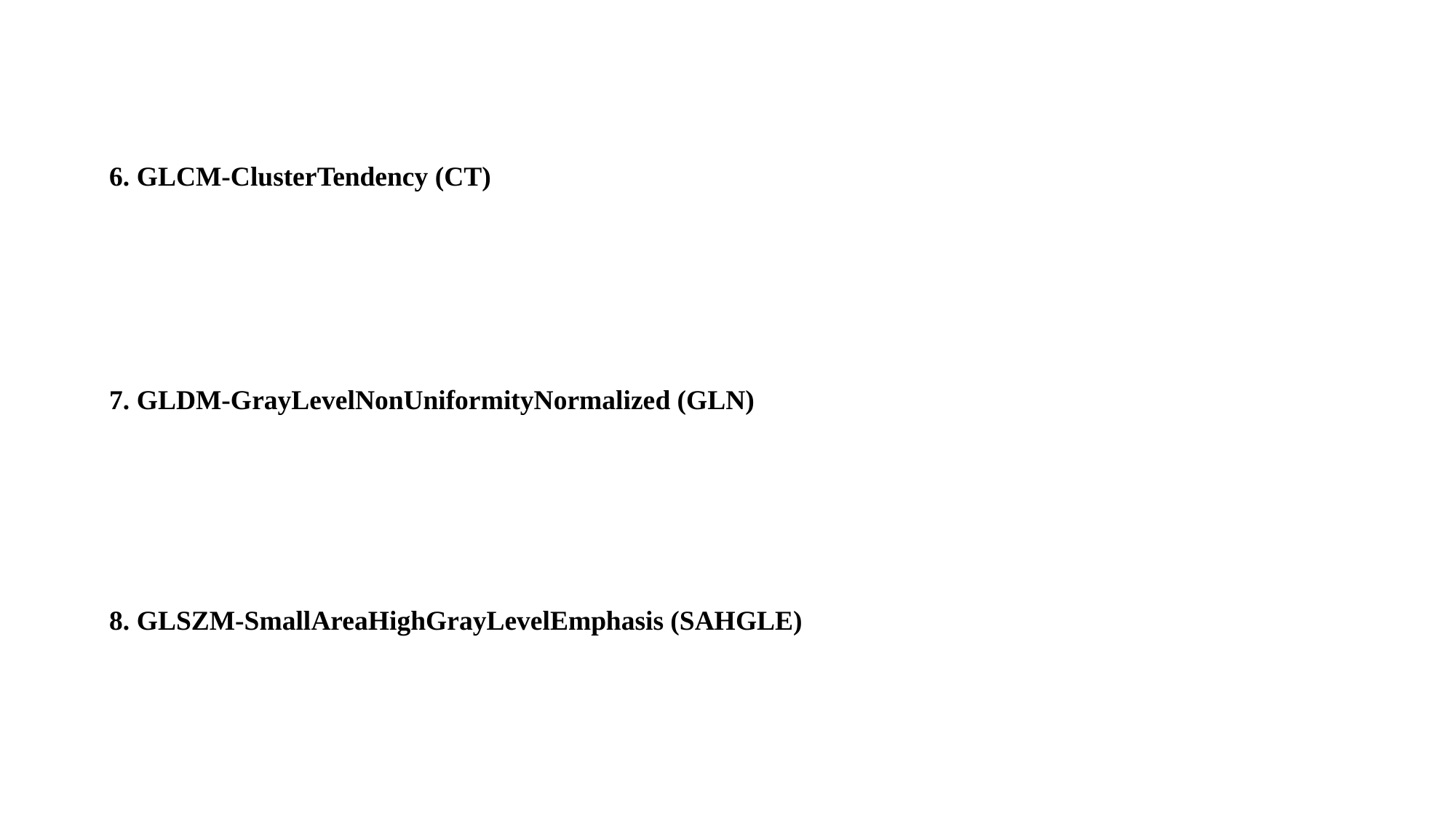

## Slide 6
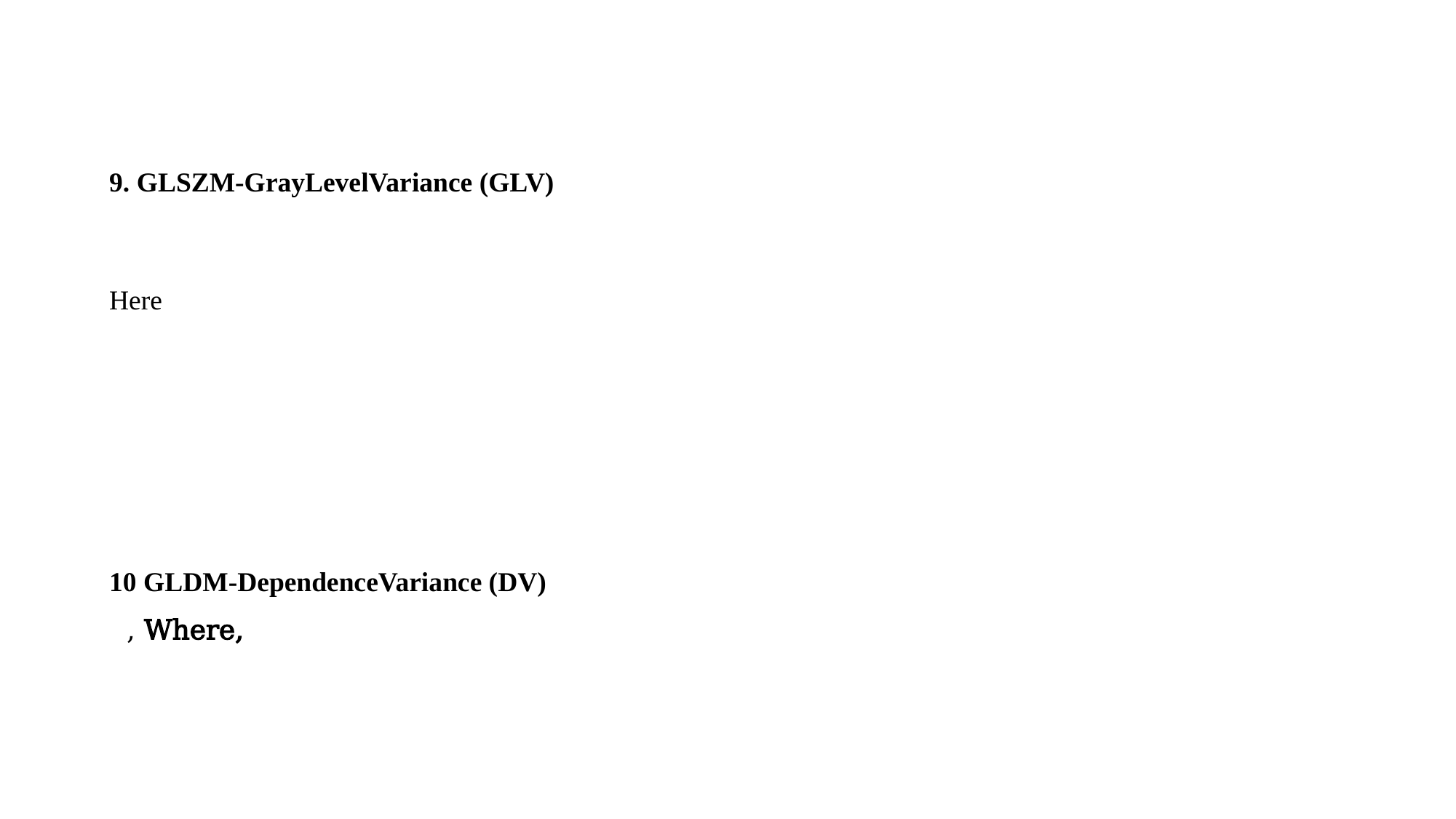

## Slide 7
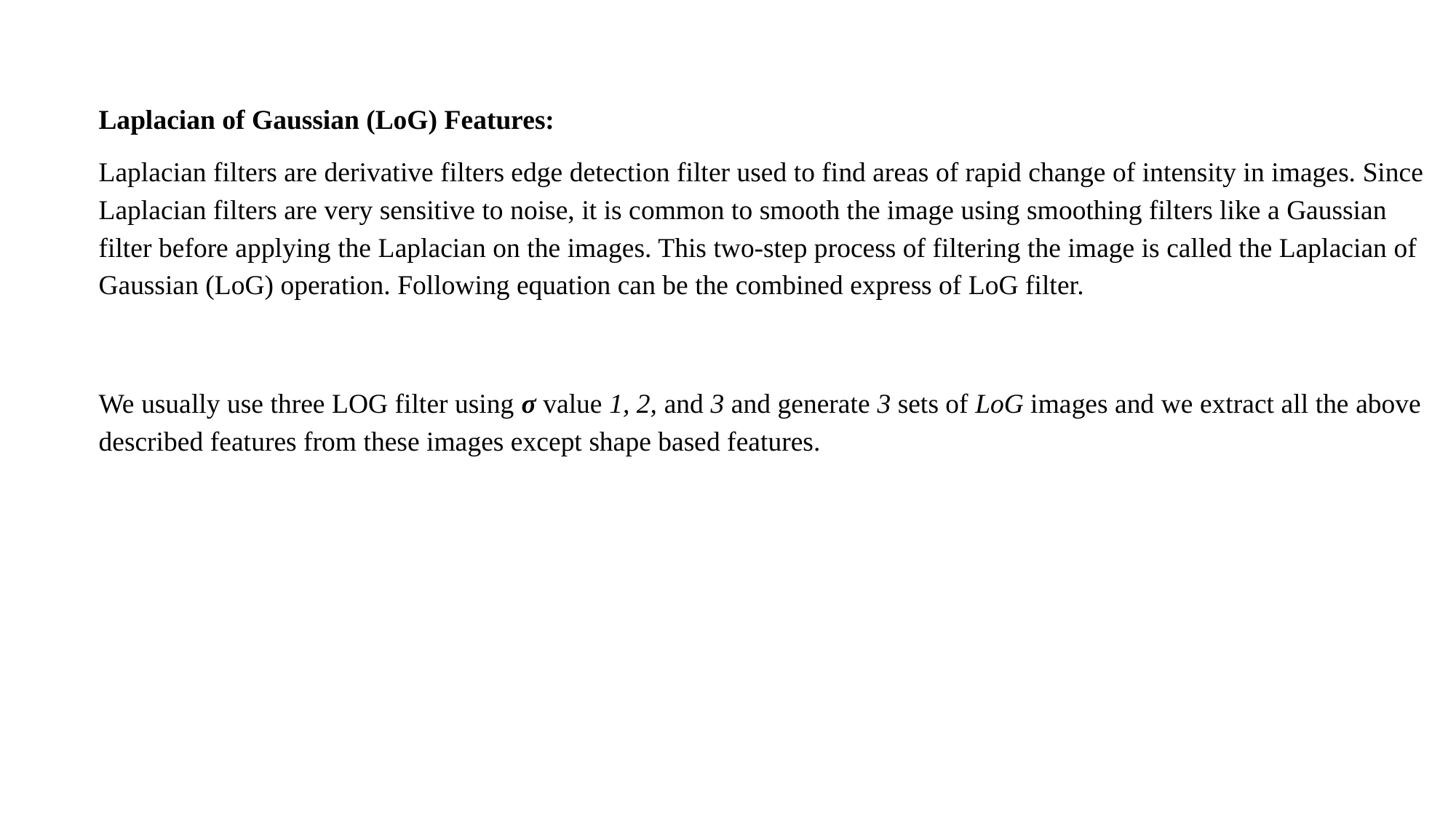

## Slide 8
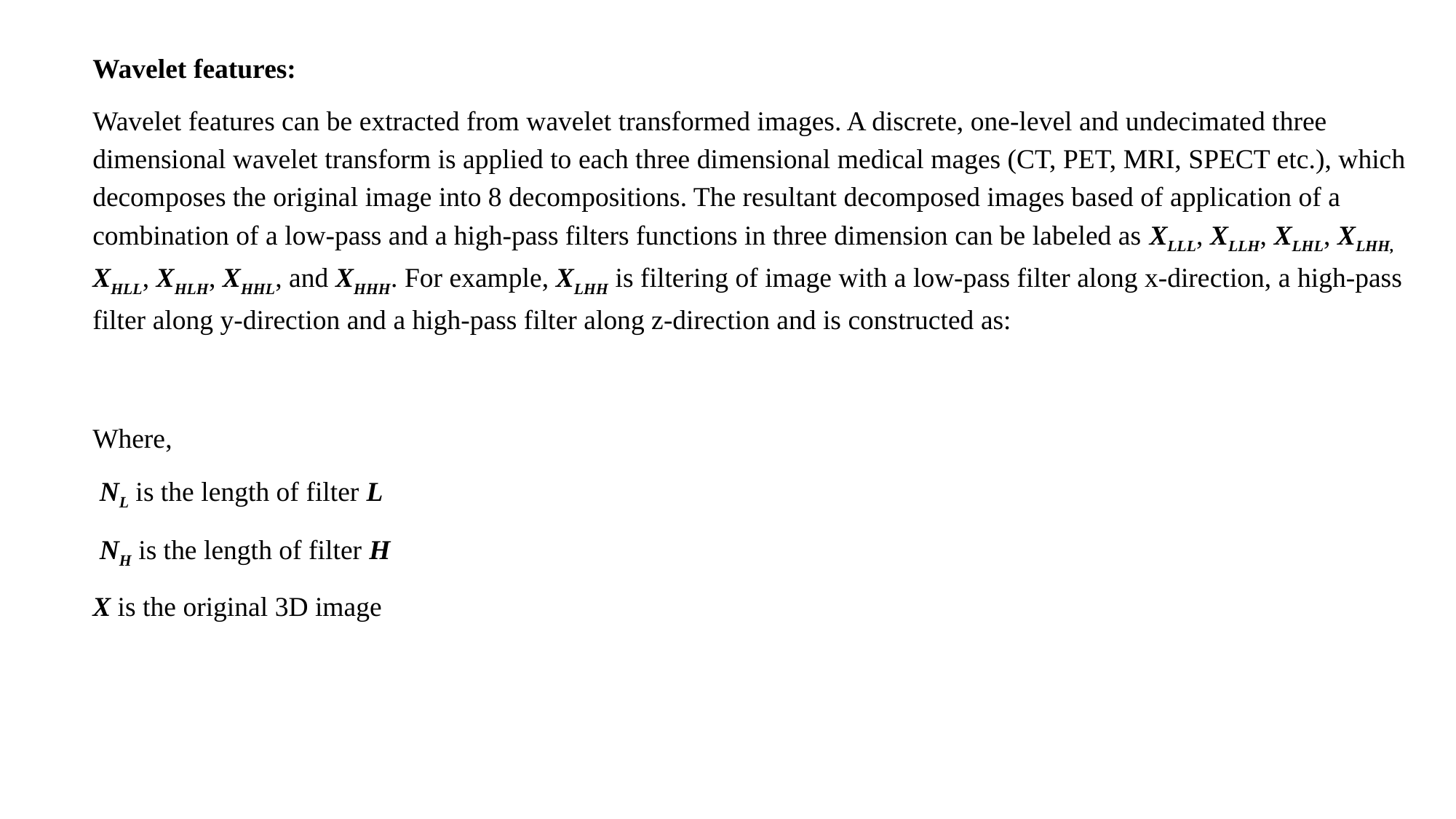

## Slide 9
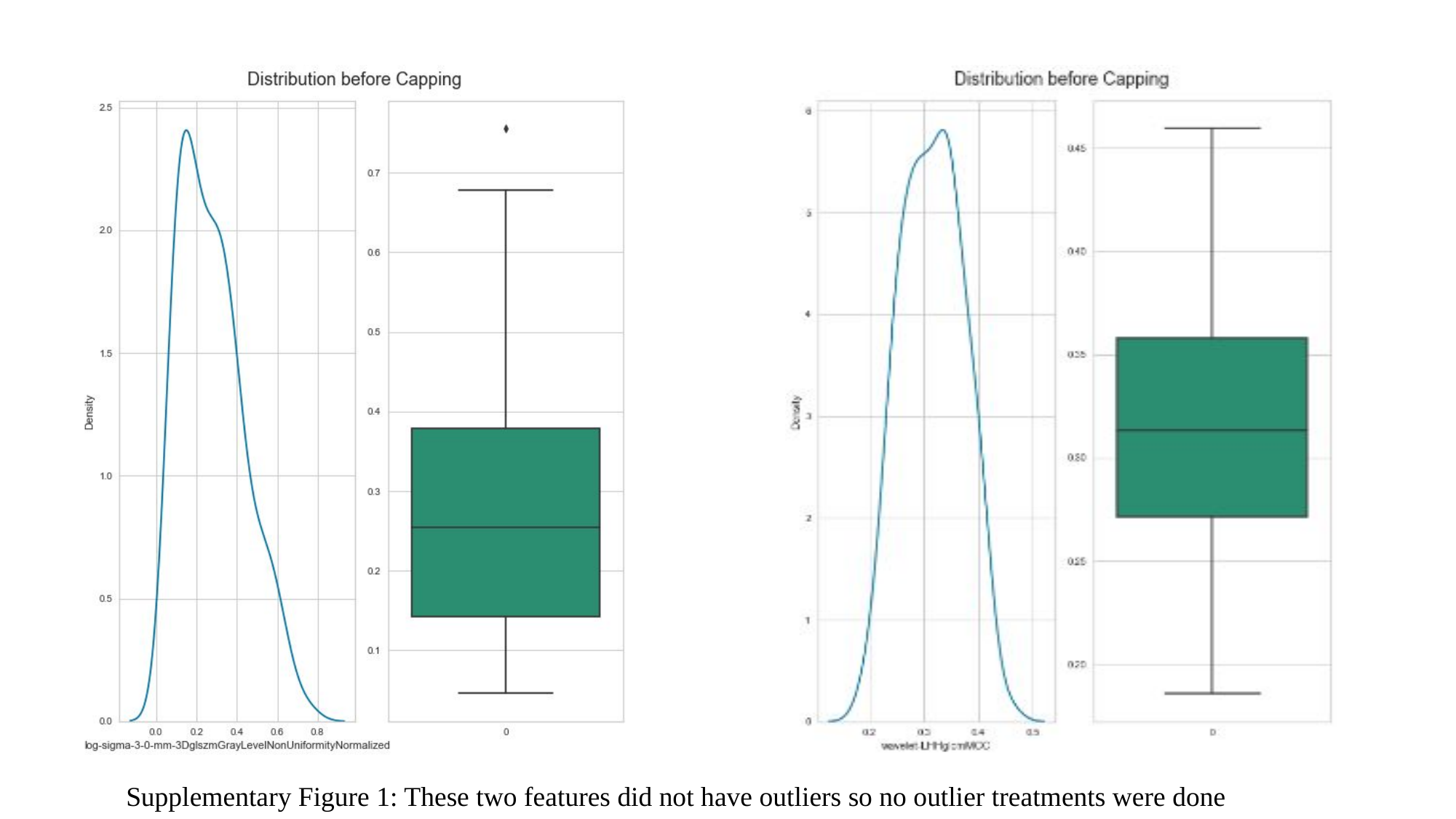

Supplementary Figure 1: These two features did not have outliers so no outlier treatments were done

## Slide 10
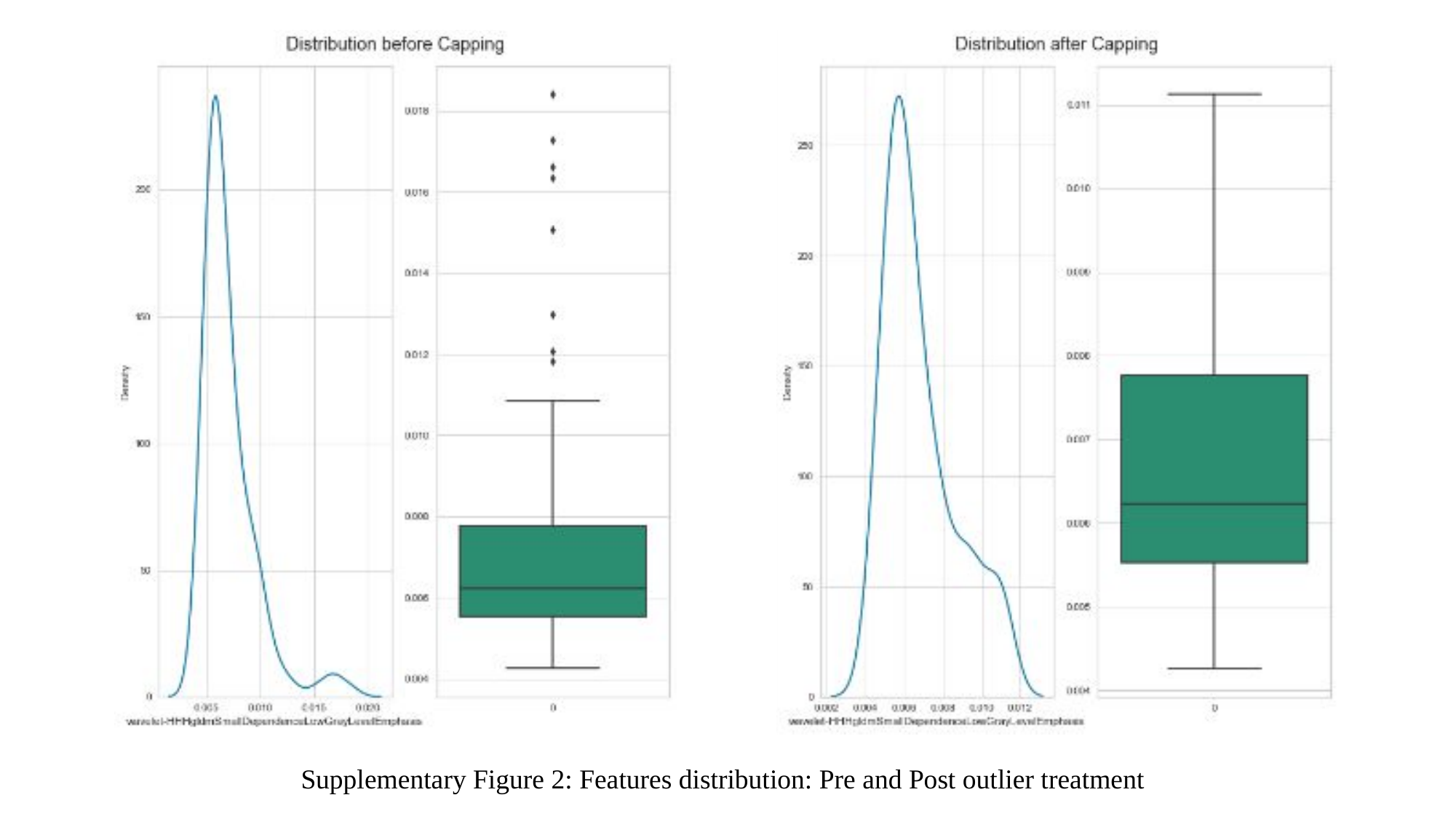

Supplementary Figure 2: Features distribution: Pre and Post outlier treatment

## Slide 11
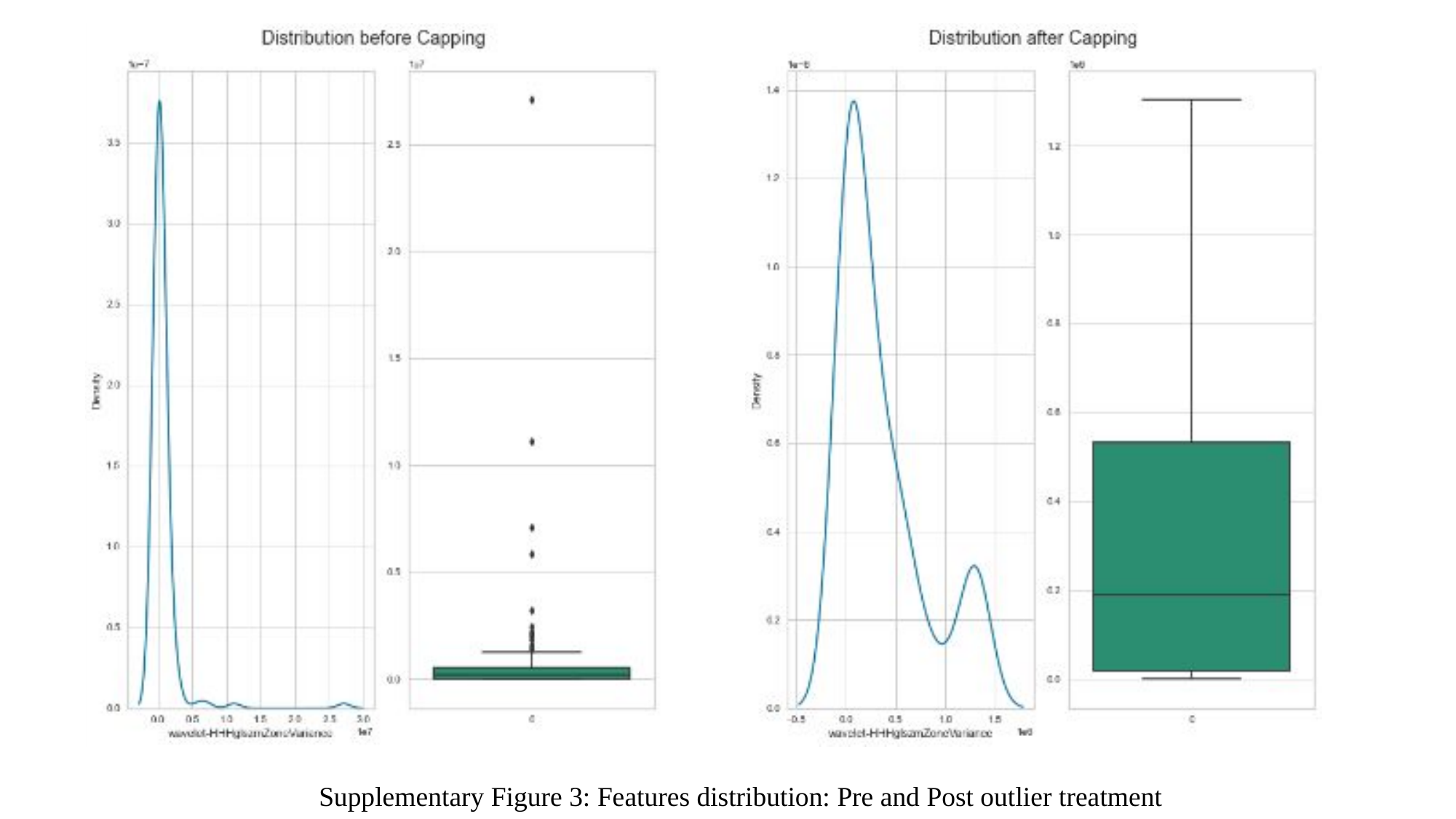

Supplementary Figure 3: Features distribution: Pre and Post outlier treatment

## Slide 12
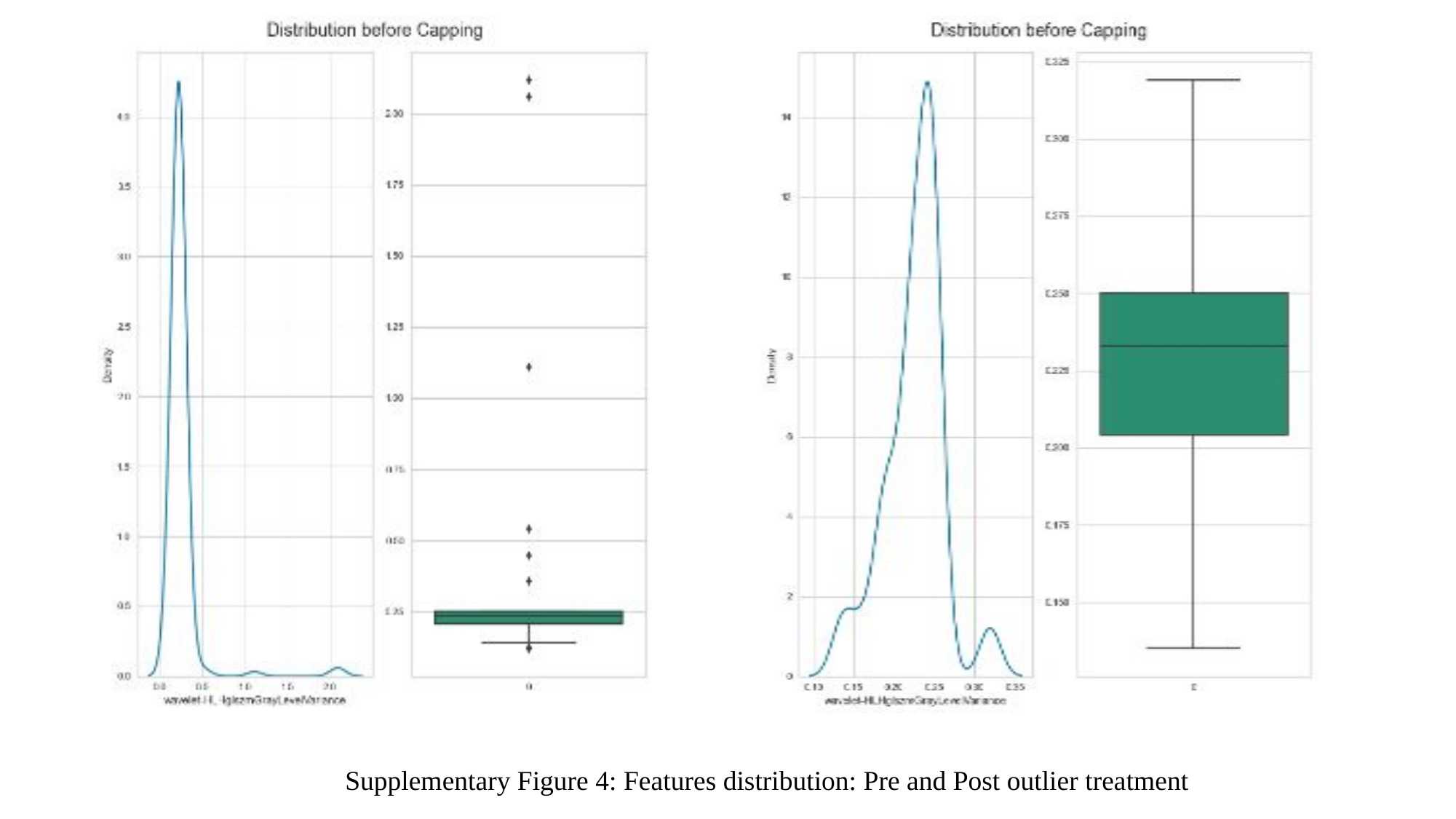

Supplementary Figure 4: Features distribution: Pre and Post outlier treatment

## Slide 13
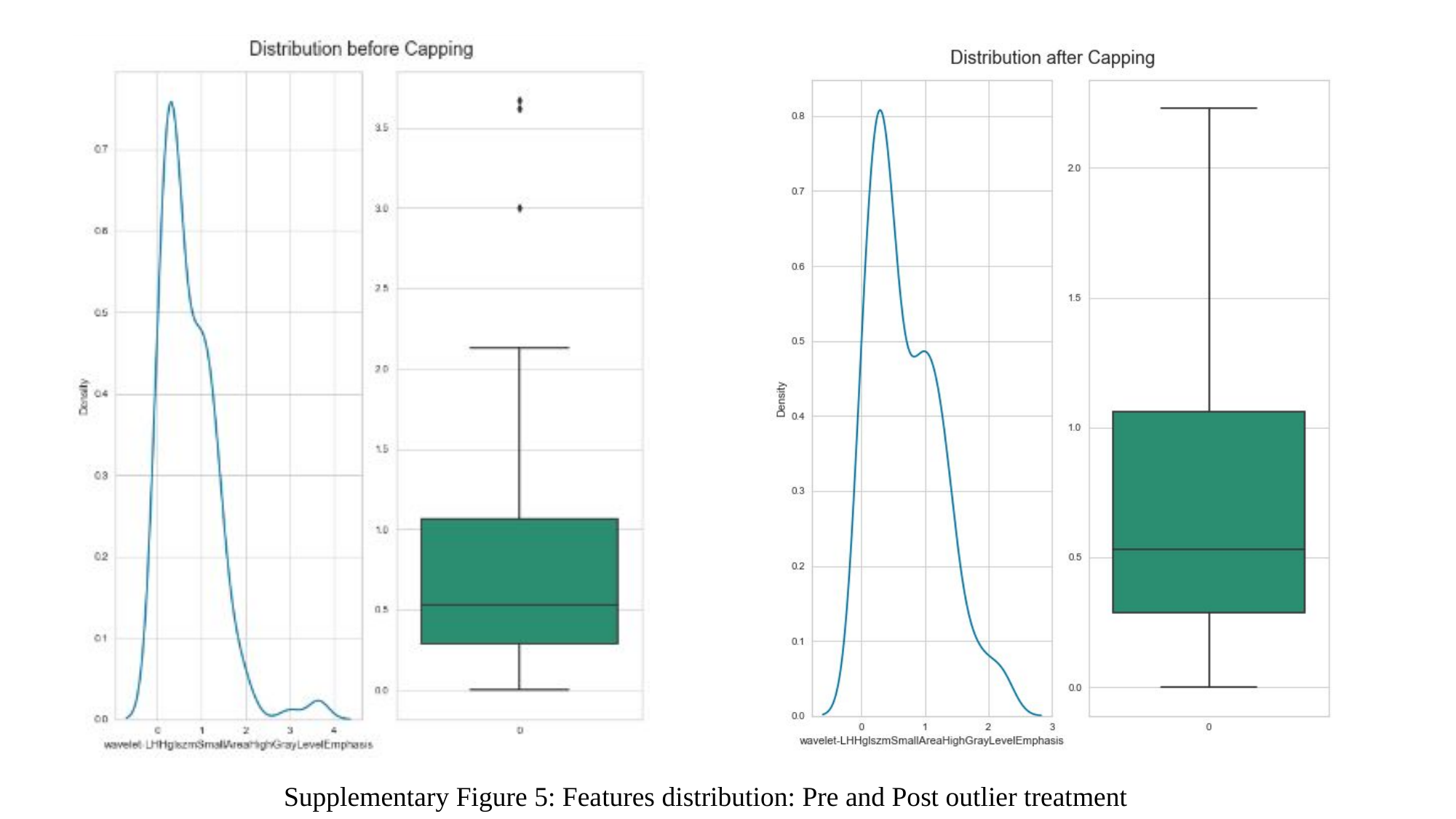

Supplementary Figure 5: Features distribution: Pre and Post outlier treatment

## Slide 14
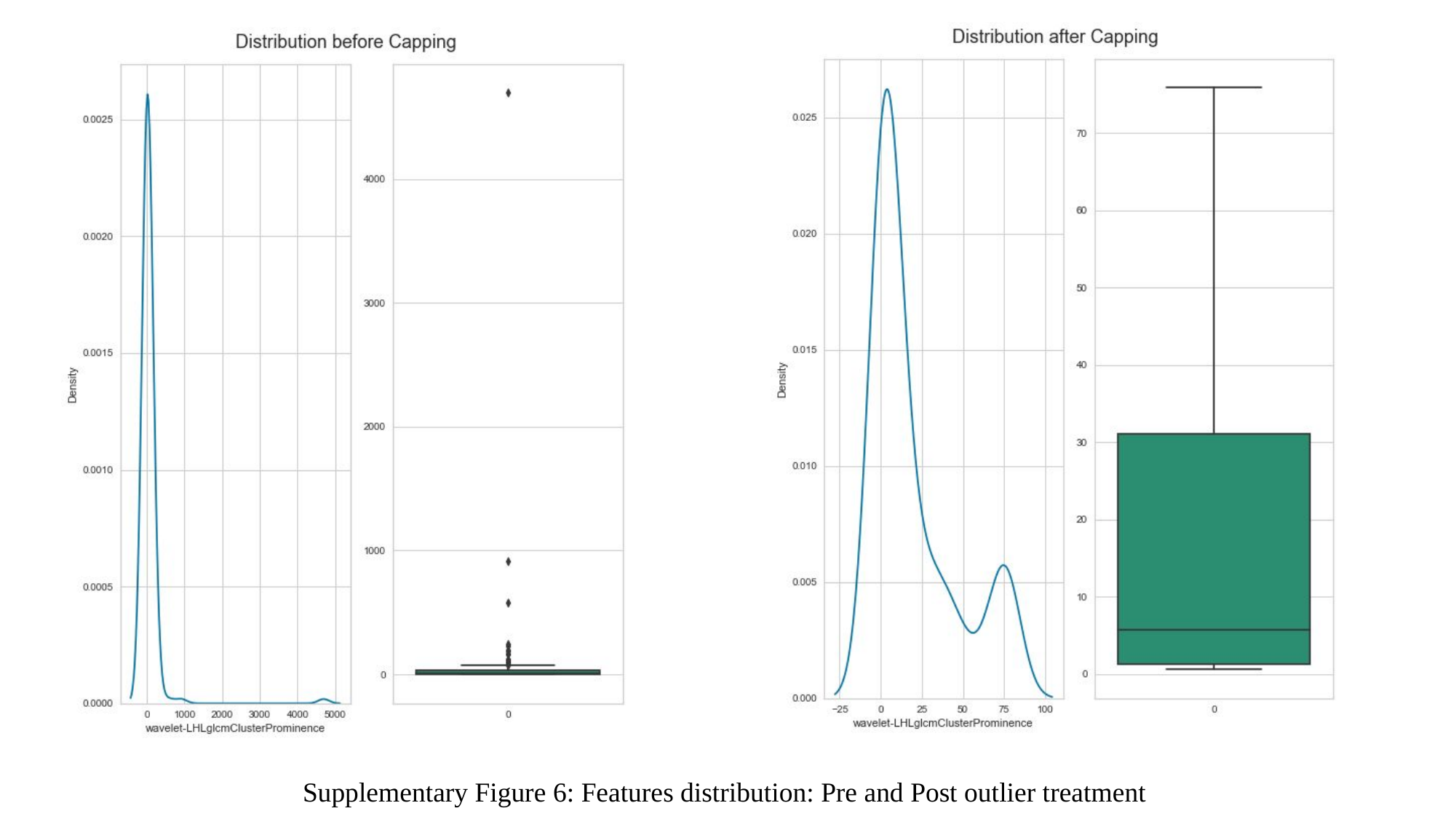

Supplementary Figure 6: Features distribution: Pre and Post outlier treatment

## Slide 15
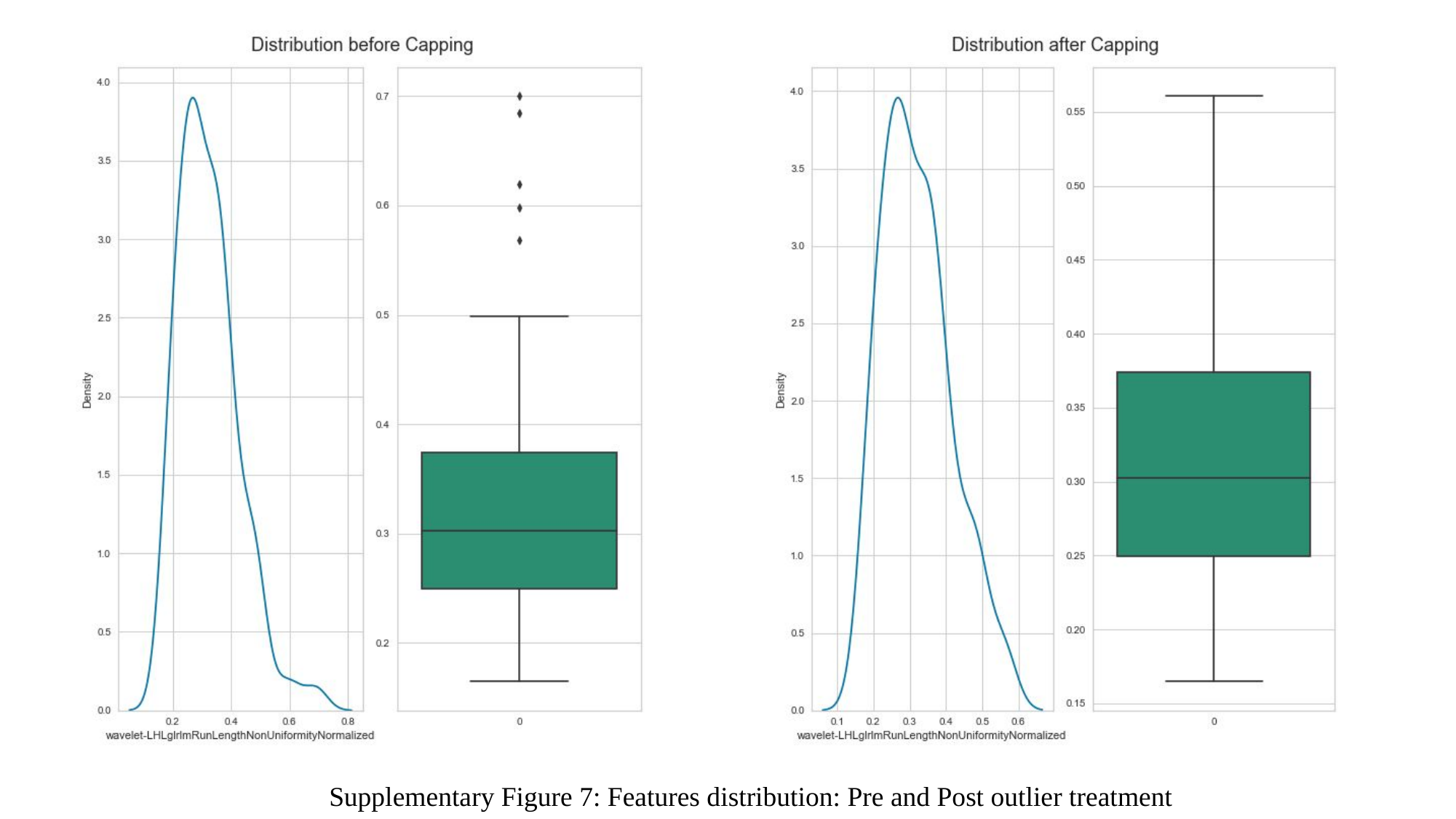

Supplementary Figure 7: Features distribution: Pre and Post outlier treatment

## Slide 16
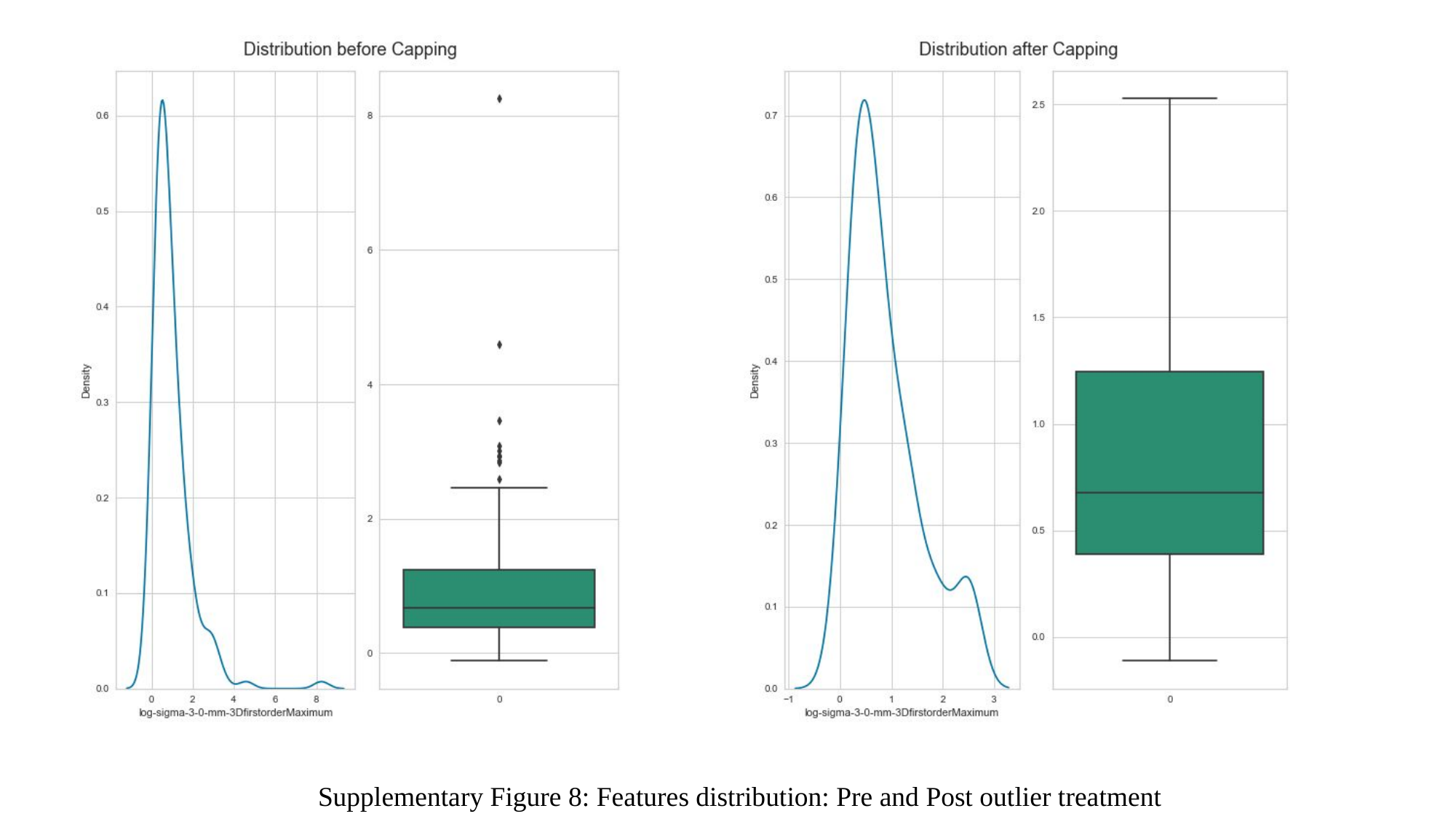

Supplementary Figure 8: Features distribution: Pre and Post outlier treatment

## Slide 17
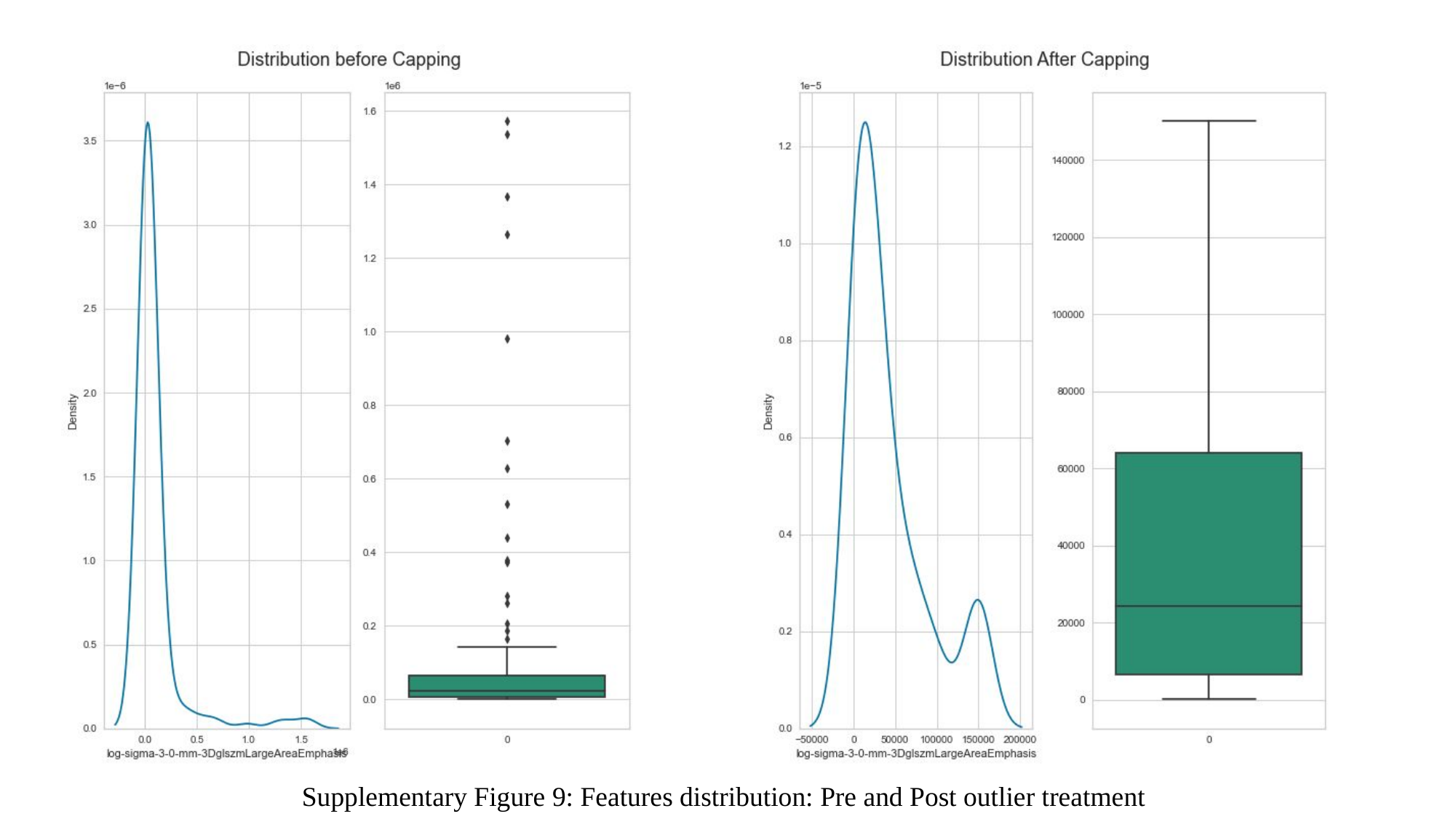

Supplementary Figure 9: Features distribution: Pre and Post outlier treatment

## Slide 18
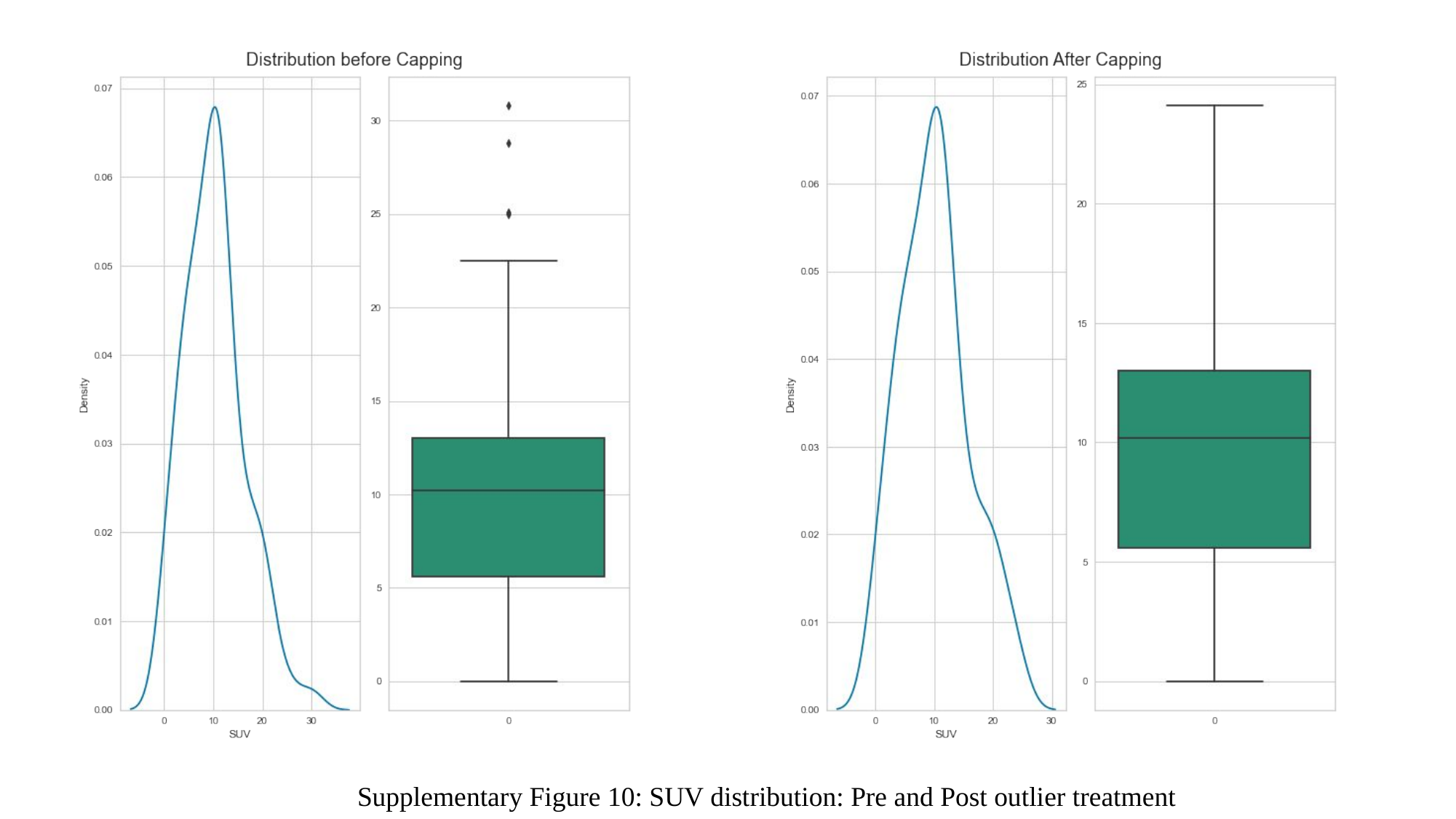

Supplementary Figure 10: SUV distribution: Pre and Post outlier treatment

## Slide 19
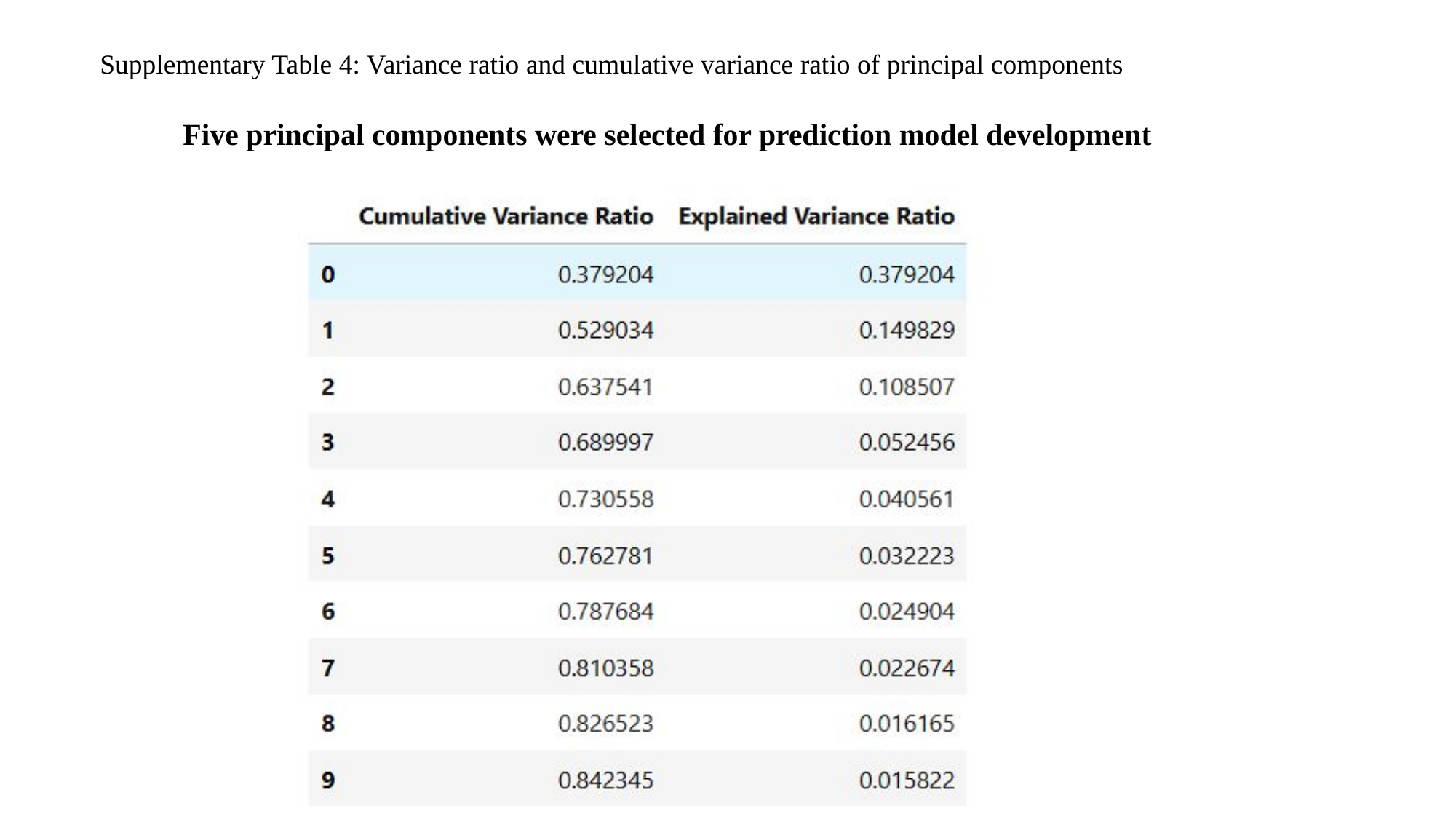

Supplementary Table 4: Variance ratio and cumulative variance ratio of principal components
Five principal components were selected for prediction model development

## Slide 20
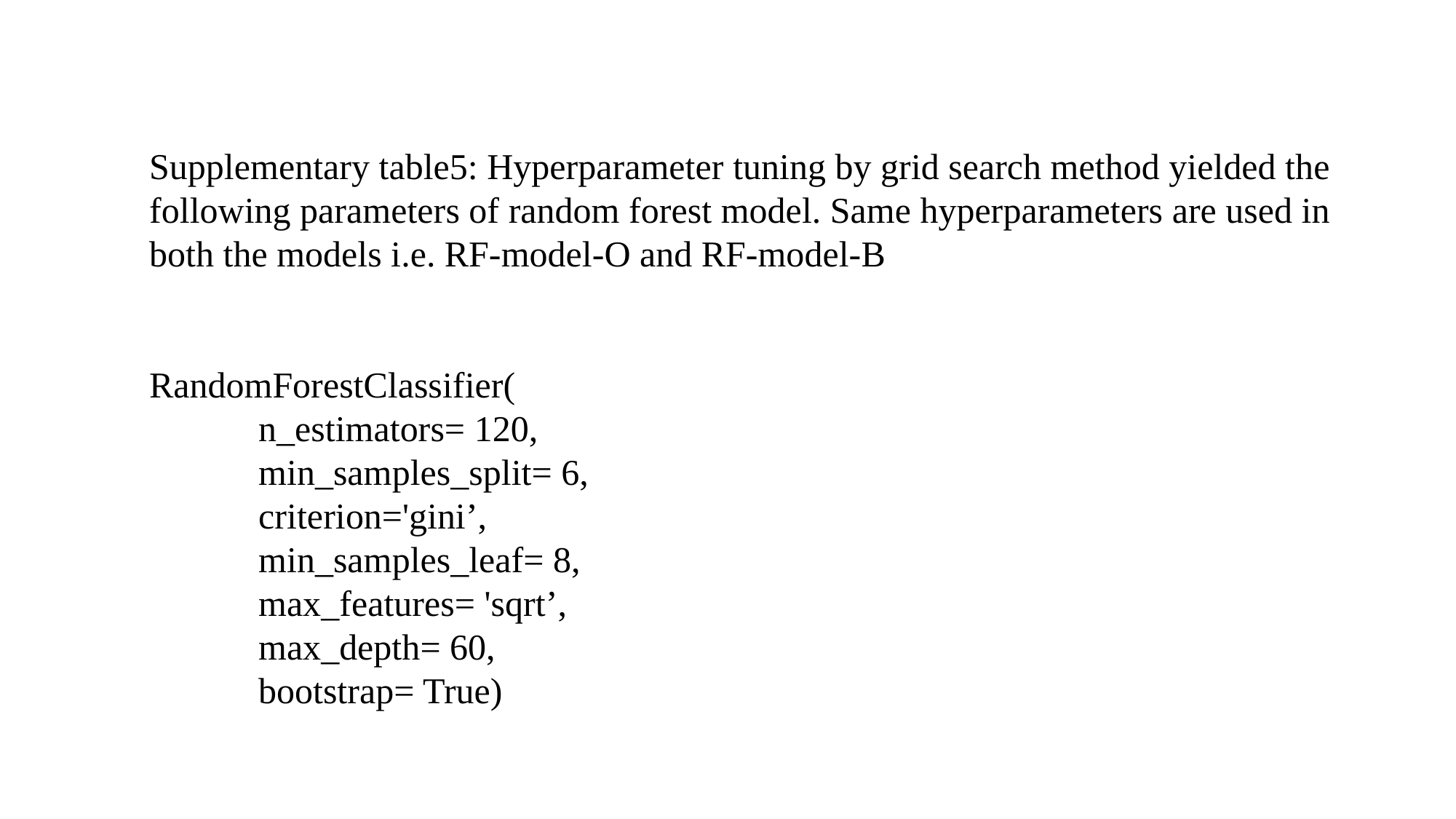

Supplementary table5: Hyperparameter tuning by grid search method yielded the following parameters of random forest model. Same hyperparameters are used in both the models i.e. RF-model-O and RF-model-B
RandomForestClassifier(
	n_estimators= 120,
	min_samples_split= 6,
	criterion='gini’,
	min_samples_leaf= 8,
	max_features= 'sqrt’,
	max_depth= 60,
	bootstrap= True)

## Slide 21
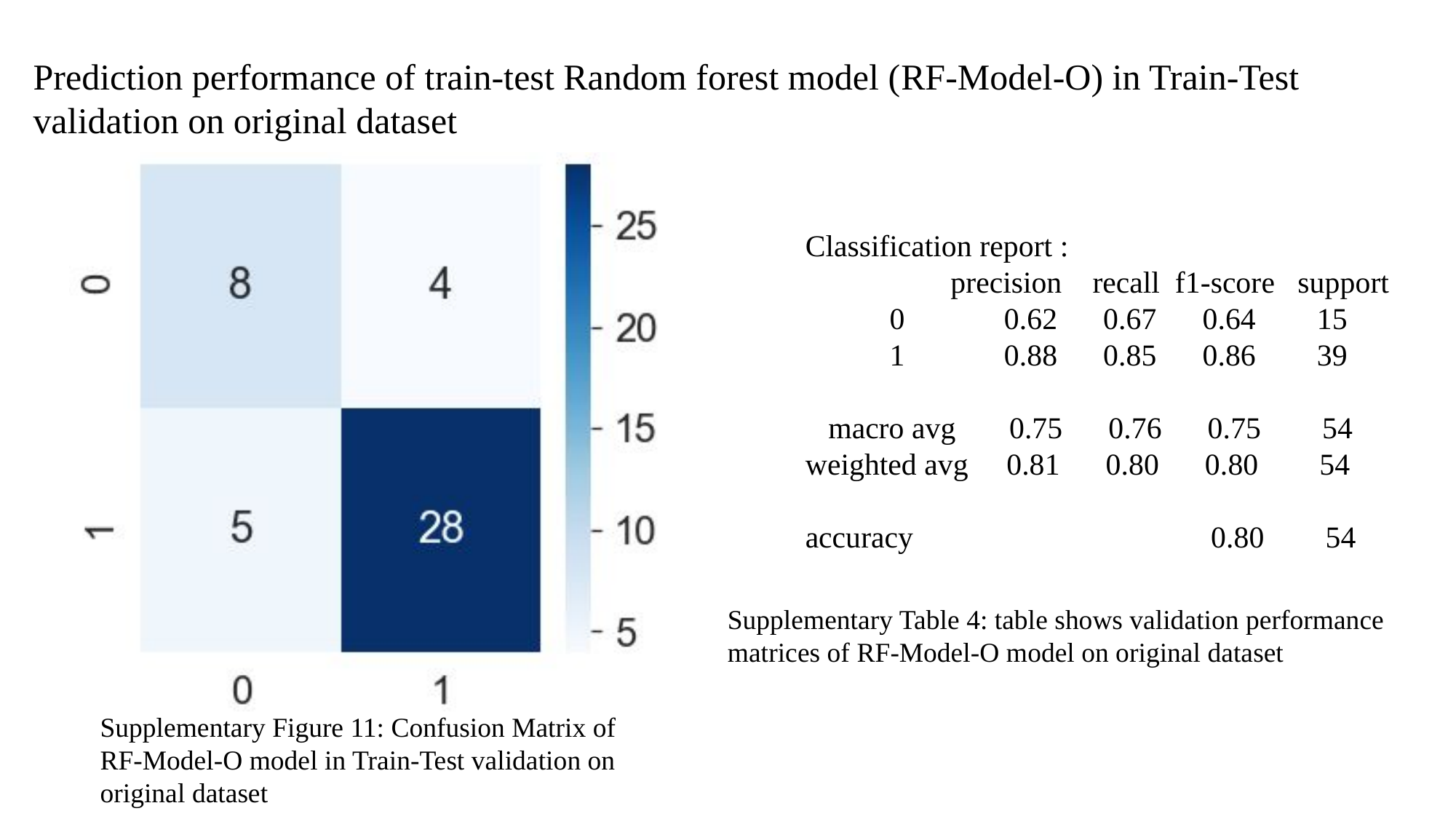

Prediction performance of train-test Random forest model (RF-Model-O) in Train-Test validation on original dataset
Classification report :
 precision recall f1-score support
 0 0.62 0.67 0.64 15
 1 0.88 0.85 0.86 39
 macro avg 0.75 0.76 0.75 54
weighted avg 0.81 0.80 0.80 54
accuracy 0.80 54
Supplementary Table 4: table shows validation performance matrices of RF-Model-O model on original dataset
Supplementary Figure 11: Confusion Matrix of RF-Model-O model in Train-Test validation on original dataset

## Slide 22
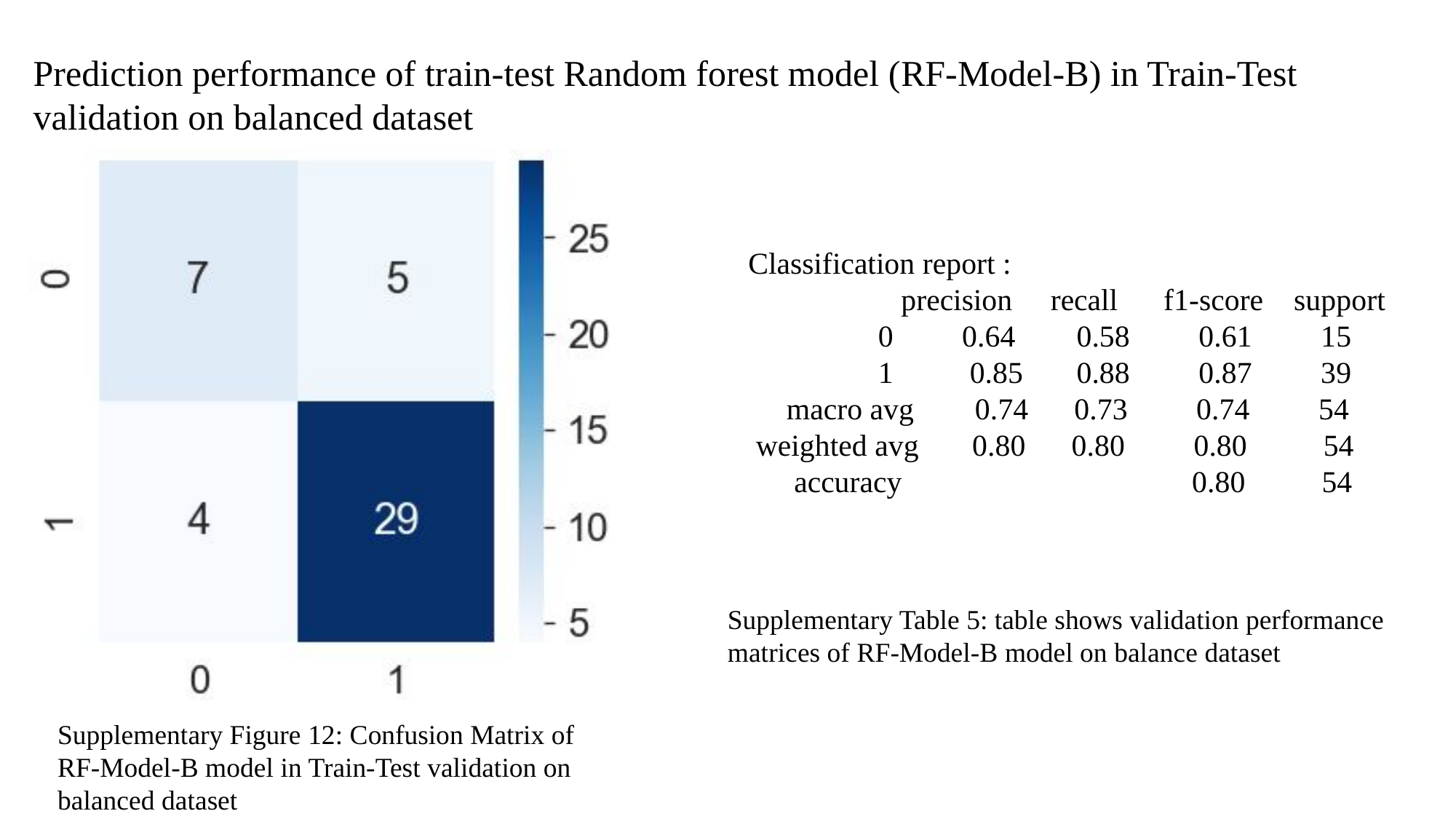

Prediction performance of train-test Random forest model (RF-Model-B) in Train-Test validation on balanced dataset
 Classification report :
 precision recall f1-score support
 0 0.64 0.58 0.61 15
 1 0.85 0.88 0.87 39
 macro avg 0.74 0.73 0.74 54
 weighted avg 0.80 0.80 0.80 54
 accuracy 0.80 54
Supplementary Table 5: table shows validation performance matrices of RF-Model-B model on balance dataset
Supplementary Figure 12: Confusion Matrix of RF-Model-B model in Train-Test validation on balanced dataset

## Slide 23
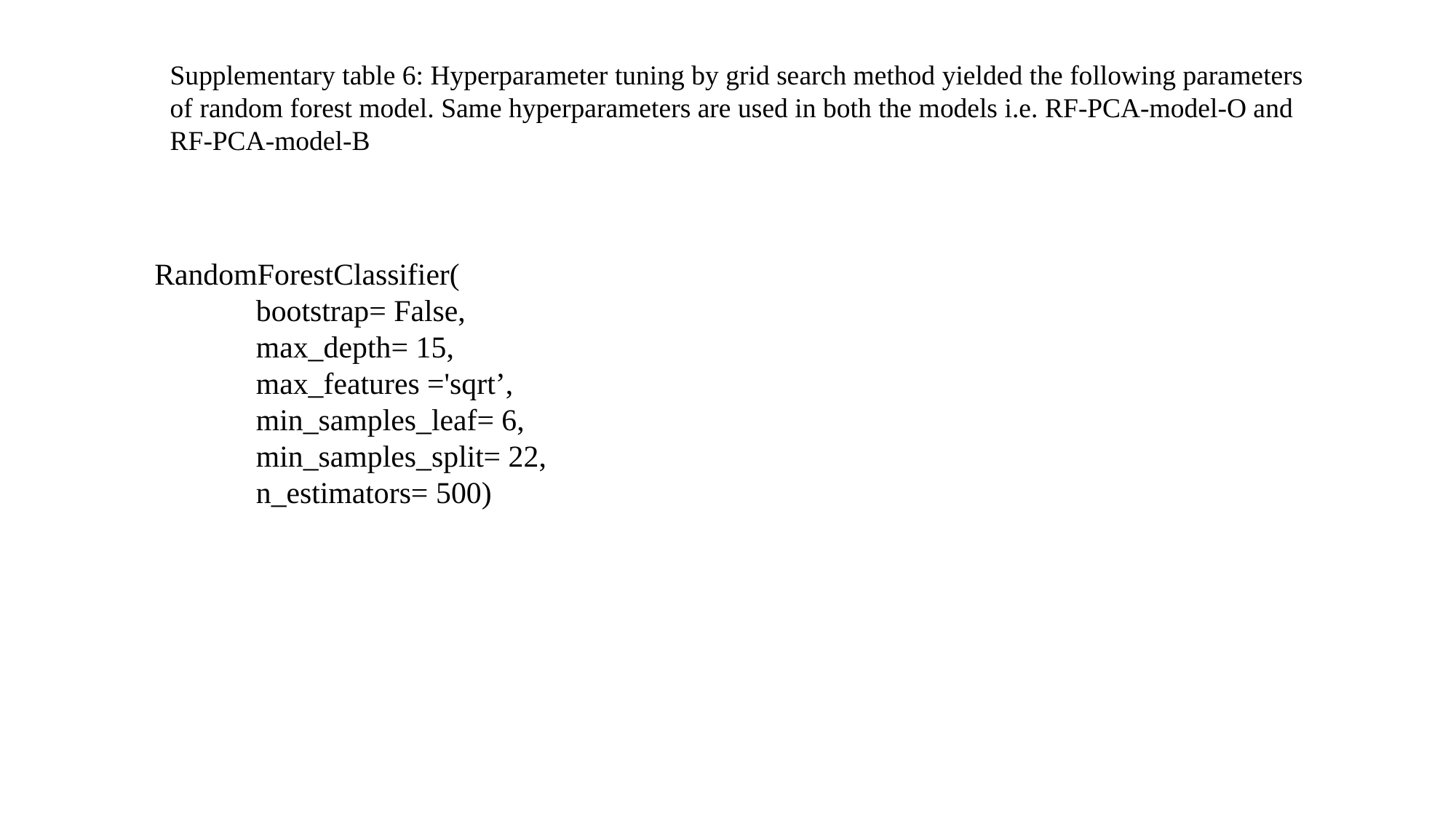

Supplementary table 6: Hyperparameter tuning by grid search method yielded the following parameters of random forest model. Same hyperparameters are used in both the models i.e. RF-PCA-model-O and RF-PCA-model-B
 RandomForestClassifier(
	bootstrap= False,
	max_depth= 15,
	max_features ='sqrt’,
	min_samples_leaf= 6,
	min_samples_split= 22,
	n_estimators= 500)
